# Future life expectancy in 35 industrialised countries: projections with a Bayesian model ensemble

**DOI:** 10.1016/S0140-6736(16)32381-9

**Published:** 2017-04-01

**Authors:** Vasilis Kontis, James E Bennett, Colin D Mathers, Guangquan Li, Kyle Foreman, Majid Ezzati

**Affiliations:** a Department of Epidemiology and Biostatistics, School of Public Health, Imperial College London, London, UK; b MRC-PHE Centre for Environment and Health, Imperial College London, London, UK; c Department of Information, Evidence and Research, World Health Organization, Geneva, Switzerland; d Department of Mathematics, Physics and Electrical Engineering, Northumbria University, Newcastle-upon-Tyne, UK; e Institute for Health Metrics and Evaluation, University of Washington, Seattle, WA, USA; f WHO Collaborating Centre on NCD Surveillance and Epidemiology, Imperial College London, London, UK

## Abstract

**Background:**

Projections of future mortality and life expectancy are needed to plan for health and social services and pensions. Our aim was to forecast national age-specific mortality and life expectancy using an approach that takes into account the uncertainty related to the choice of forecasting model.

**Methods:**

We developed an ensemble of 21 forecasting models, all of which probabilistically contributed towards the final projections. We applied this approach to project age-specific mortality to 2030 in 35 industrialised countries with high-quality vital statistics data. We used age-specific death rates to calculate life expectancy at birth and at age 65 years, and probability of dying before age 70 years, with life table methods.

**Findings:**

Life expectancy is projected to increase in all 35 countries with a probability of at least 65% for women and 85% for men. There is a 90% probability that life expectancy at birth among South Korean women in 2030 will be higher than 86·7 years, the same as the highest worldwide life expectancy in 2012, and a 57% probability that it will be higher than 90 years. Projected female life expectancy in South Korea is followed by those in France, Spain, and Japan. There is a greater than 95% probability that life expectancy at birth among men in South Korea, Australia, and Switzerland will surpass 80 years in 2030, and a greater than 27% probability that it will surpass 85 years. Of the countries studied, the USA, Japan, Sweden, Greece, Macedonia, and Serbia have some of the lowest projected life expectancy gains for both men and women. The female life expectancy advantage over men is likely to shrink by 2030 in every country except Mexico, where female life expectancy is predicted to increase more than male life expectancy, and in Chile, France, and Greece where the two sexes will see similar gains. More than half of the projected gains in life expectancy at birth in women will be due to enhanced longevity above age 65 years.

**Interpretation:**

There is more than a 50% probability that by 2030, national female life expectancy will break the 90 year barrier, a level that was deemed unattainable by some at the turn of the 21st century. Our projections show continued increases in longevity, and the need for careful planning for health and social services and pensions.

**Funding:**

UK Medical Research Council and US Environmental Protection Agency.

## Introduction

In high-income countries, except in periods of war, famine, and infection outbreaks, national life expectancy has increased steadily for decades, although there has been stagnation or decline in poor and marginalised groups.^1^, ^2^, ^3^ At the same time, due to variations in the pace of increase, the country with the highest (frontier) life expectancy has changed several times.^3^

Projections of future mortality and life expectancy are needed to plan for health and social services and pensions. Most current mortality and life expectancy projections rely on a single model, selected based on either theoretical considerations or a comparison of a few models.^4^ Due to differences in methodology, such projections vary across studies. For example, some researchers have projected a continued rise in life expectancy in high-income countries.^3^, ^5^ Others have argued that obesity and other risks to health will imminently (ie, within the first half of the 21st century) halt or reverse the rise in life expectancy.^6^ This discrepancy indicates that there is uncertainty about model choice, which is not taken into account when a single model is used.

We applied a probabilistic Bayesian model averaging (BMA) approach to mortality and life expectancy projection.^7^ The BMA approach, which is increasingly common in areas of science such as weather and climate forecasting, uses an ensemble of models, all of which probabilistically contribute towards the final projections.

## Methods

### Countries analysed

Our analysis covered high-income countries in Asia and the Pacific, North America, central Europe and western Europe, as well as Latin American countries that are members of the Organisation for Economic Co-operation and Development (OECD), with data available on deaths and population from 1985 to 2010 or later, and a population of 1 million or more over the entire period. Having data from at least 1985 provided a sufficient number of years to estimate parameters and weights for the models as described below. The table lists the countries together with details about data availability.

**Research in context**

**Evidence before this study**

We searched PubMed for articles published up to July 21, 2016, with no language restrictions, using the search terms “forecasting” OR “projection”, “Bayesian”, and “life expectancy” in the publication title and abstract. We also used a review of forecasting methods to identify previous studies. All identified studies had used a single preferred model for projecting mortality or life expectancy or both for one or multiple countries.

**Added value of this study**

In a novel approach, we used an ensemble of models, all of which probabilistically contribute toward the final projections, with the projections from each model weighted according to how well it predicted withheld data for each country and sex. This approach not only takes into account the inherent uncertainty in the choice of projection model, but also outperforms projections from the single-model approach. We also decomposed the projected rise in life expectancy into gains in younger, middle, and older ages.

**Implications of all the available evidence**

There is a high probability that life expectancy will continue to increase in all industrialised countries, and more than a 50% chance that by 2030 national female life expectancy in South Korea will break the 90 year barrier. Enhanced longevity in older ages will be the main contributor to the projected gains in life expectancy at birth, especially in women.

### Data sources

We obtained data on population and deaths from WHO. We obtained data for a single year (2014) for South Korea from the South Korean Statistical Information Service (KOSIS) because the latest year reported to WHO was 2013 at the time of analysis. All countries had data from 1985 onwards; 24 countries had data from 1960 onwards and were used for testing the performance of BMA projections. Information about data pre-processing is described in the appendix (p 3).

### Bayesian model averaging

The probabilistic BMA approach uses an ensemble of models, each providing a posterior distribution for life expectancy in the future. The posterior distribution of the final projections is a probabilistic combination of those from individual models.^7^

We used an ensemble of 21 models for projecting age-specific death rates, which were in turn used to calculate projected life expectancy. These models, described in detail in the appendix (pp 4–6), were formulated to incorporate features of death rates in relation to age and birth cohort, and over time, as well as statistical considerations such as extent of smoothing over age and birth cohort, and how much weight to give to older data-points compared with more recent ones. We determined which models received a greater or lesser weight in the final projections based on performance in projecting withheld data in the following steps.

In step 1, we measured the performance of projections from individual models. We held back the last 13 years of data for each country (table), and used the remaining data to estimate model parameters, which we then used to make projections for the withheld years; 13 years is the maximum duration that would still allow sufficient data to reliably estimate model parameters for countries whose data start in 1985. We used bias in life expectancy, calculated as described in the appendix (p 7), as the metric to measure projection error for reasons described in the appendix (p 10). We calculated projection bias as the difference between the observed (but withheld) and projected life expectancies, averaged over all years of withheld data.

In step 2, we calculated model weights such that models with smaller bias were assigned larger weights, with the weights decaying exponentially as the magnitude of the projection bias increased. Each model was assigned a weight of exp(–|Projection bias|), where the bias was calculated as described in step 1; the 21 weights were normalised to sum to 1. The model weights are shown in the appendix (pp 16, 17).

In step 3, we calculated the final projections. We used all the available data for each country and sex to estimate model parameters, which we used to make projections to 2030. We took a number of draws from the posterior distribution of age-specific death rates under each model proportional to the weight of that model calculated in step 2, and pooled the draws to obtain the posterior distribution of age-specific death rates under the BMA, which were then used to calculate life expectancy projections. The code used for the analysis is available online.

We tested the performance of the BMA projections as described in the appendix (pp 8, 9), which showed that the BMA approach on average had a smaller error than the best single models for different sex, country, and projection duration combinations (appendix pp 18, 19). The relative advantage of the BMA compared with the best model increased for longer validation periods. For a projection duration of 22 years, the average absolute bias of the BMA across countries and years was 0·68 years for women and 1·09 years for men; that of the best model was 1·00 years for women and 1·39 years for men. The 90% coverage of the BMA projection uncertainty, which measures how well the posterior distributions of projected life expectancies coincide with withheld data, was on average 90·2% for women and 83·9% for men, indicating a slight underestimation of coverage for men (appendix p 35).

### Role of the funding source

The funder had no role in study design, data collection and analysis, interpretation, or writing of the report. VK, JEB, and CDM had full access to all data used in this study. ME was responsible for submitting the Article for publication.

## Results

**Figure 1:**
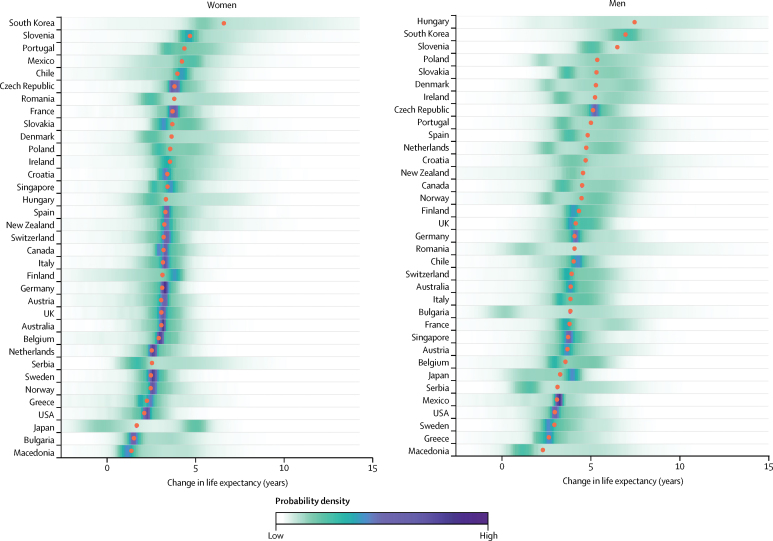
Posterior distribution of projected change in life expectancy at birth from 2010 to 2030 Red dots show the posterior medians. Countries are ordered vertically by median projected increase from largest (at the top) to smallest (at the bottom).

Figure 1 shows the projected change in life expectancy at birth from 2010 to 2030. Taking model uncertainty into account, we project that life expectancy will increase in all of these 35 countries with a probability of at least 65% for women and 85% for men, although the increase will vary across countries. There is nonetheless a 35% probability that life expectancy will stagnate or decrease in Japanese women by 2030, followed by a 14% probability in Bulgarian men and 11% in Finnish women.

**Figure 2:**
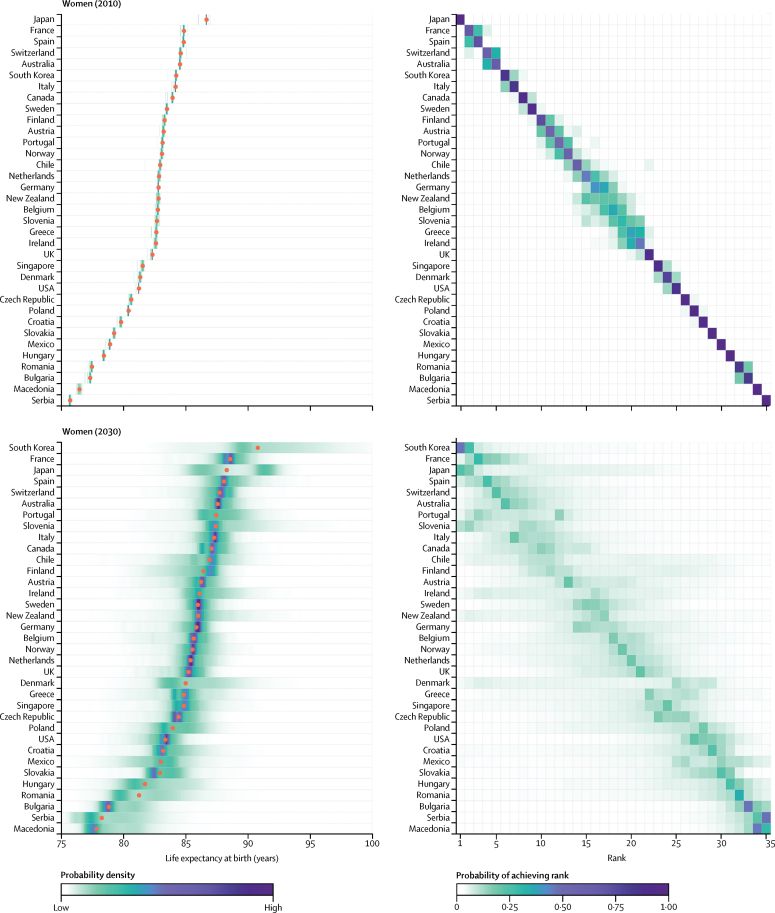
Women's life expectancy at birth in 2010 and 2030 (Left) Posterior distribution of life expectancy and its median value. Red dots show the posterior medians. (Right) Probability distribution for the country's rank. See the appendix (p 36) for numerical values. Countries are ordered vertically by median life expectancy from largest (at the top) to smallest (at the bottom).

**Figure 3:**
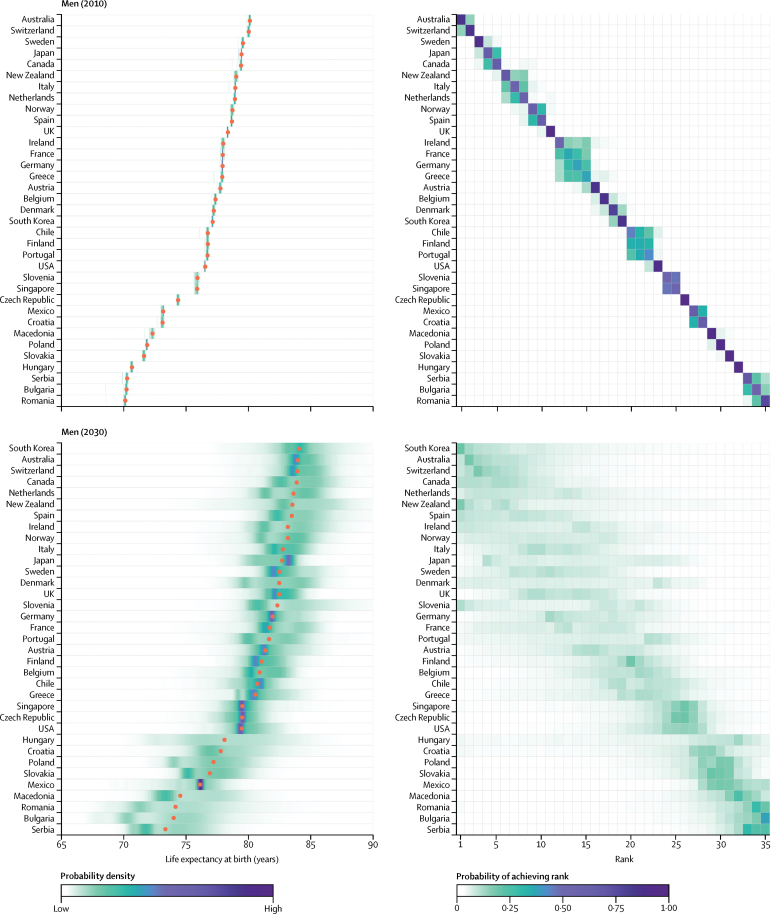
Men's life expectancy at birth in 2010 and 2030 (Left) Posterior distribution of life expectancy and its median value. Red dots show the posterior medians. (Right) Probability distribution for the country's rank. See the appendix (p 36) for numerical values. Countries are ordered vertically by median life expectancy from largest (at the top) to smallest (at the bottom).

Figure 2, Figure 3 show the distributions of projected life expectancy at birth for women and men by country, and the distribution of each country's rank. There is 90% probability that life expectancy at birth among South Korean women in 2030 will be higher than 86·7 years, the same as the highest life expectancy in the world in 2012, and a 57% probability that it will be higher than 90 years (figure 2), a level that was considered virtually unattainable at the turn of the 21st century by some researchers.^8^, ^9^, ^10^ This achievement is a continuation of the massive gains in South Korean women's life expectancy, which has increased by on average 3·7 years per decade since 1985, when they were ranked 29th, with no indication of slowing (appendix pp 20–27). The probability that South Korean women will have the highest female life expectancy in 2030 is 45%, with a 27% probability of being in second place.

Female life expectancy in South Korea is followed by those in France, Japan, and Spain, the projected distributions of which have substantial overlap. For example, although median projected life expectancy for Japan places it in third place in 2030, there is a 22% probability that Japanese women will continue to have the highest female life expectancy in 2030, as they have had for decades, and 14% probability of being ranked second. Other countries with large projected gains in female longevity are emerging economies (eg, Slovenia) as well as Portugal and France. Slovenia and Portugal have 71% and 63% probabilities, respectively, to be in the top ten countries in 2030, a substantial improvement to their rankings in the 1980s.

**Figure 4:**
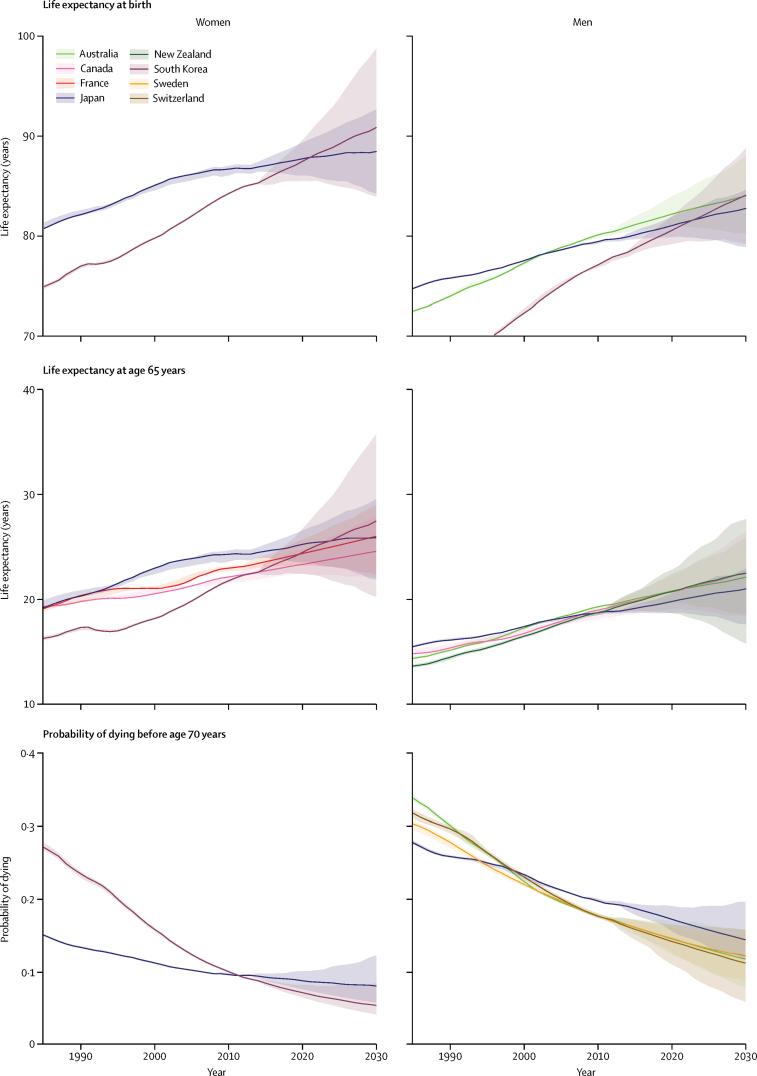
Trends in life expectancy at birth, life expectancy at age 65 years, and the probability of dying before age 70 years in countries that have attained, or are projected to attain, the highest life expectancy or lowest probability of dying before age 70 years in at least 1 year from 1985 to 2030

For men, South Korea, Australia, and Switzerland have highly overlapping distributions of projected life expectancy and hence similar probabilities of occupying the top three ranks (figure 3). There is an at least 95% probability that men's life expectancy at birth in these three countries will surpass 80 years in 2030, and 27% that it would surpass 85 years. Like women, men in South Korea and a number of central European emerging economies like Hungry and Slovenia are projected to make large gains, as are men in Denmark, Ireland, and a few other western European nations. As a result of these trends, by 2030, South Korea is likely to take the life expectancy frontier position from Japan for women, and catch up with the current global frontier country Australia for men (figure 4). The USA, Japan, Sweden, Greece, Macedonia, and Serbia have some of the lowest projected gains for both men and women.

**Figure 5:**
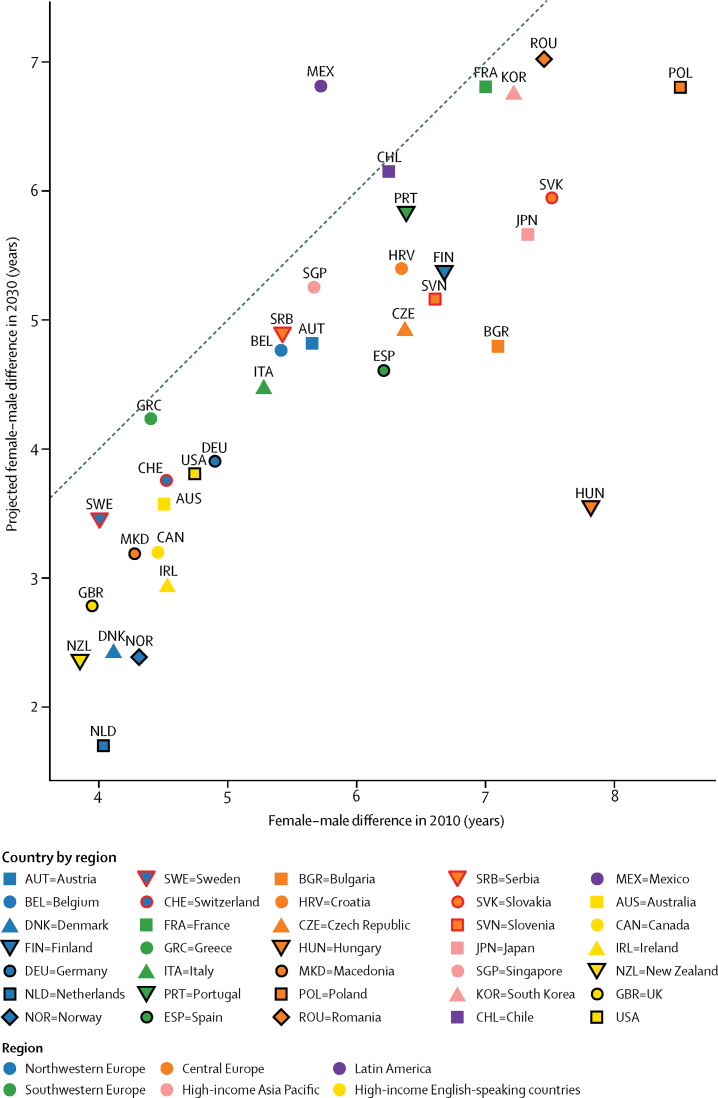
Life expectancy difference between women and men in 2030 versus 2010 The points show the difference of posterior median life expectancy at birth between women and men in 2030 and 2010. The colour of each point denotes the country's geographical region.

In 2010, women had a higher life expectancy than men by 3·9 (New Zealand) to 8·5 (Poland) years (figure 5). We project that the female advantage will shrink by 2030 in every country analysed here except Mexico, where a slightly larger life expectancy gain is projected for women than for men, and in Chile, France, and Greece where male and female life expectancies will increase by about the same.

**Figure 6:**
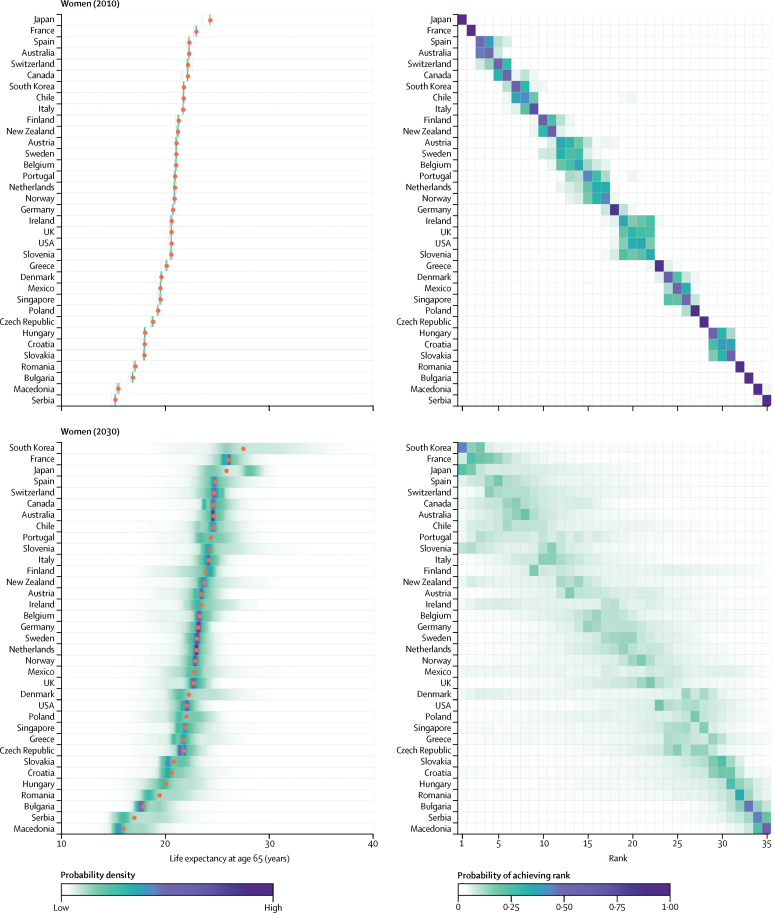
Women's life expectancy at age 65 years in 2010 and 2030 (Left) Posterior distribution of life expectancy at age 65 years and its median value. Red dots show the posterior medians. (Right) Probability distribution for the country's rank. See the appendix (p 37) for numerical values.

**Figure 7:**
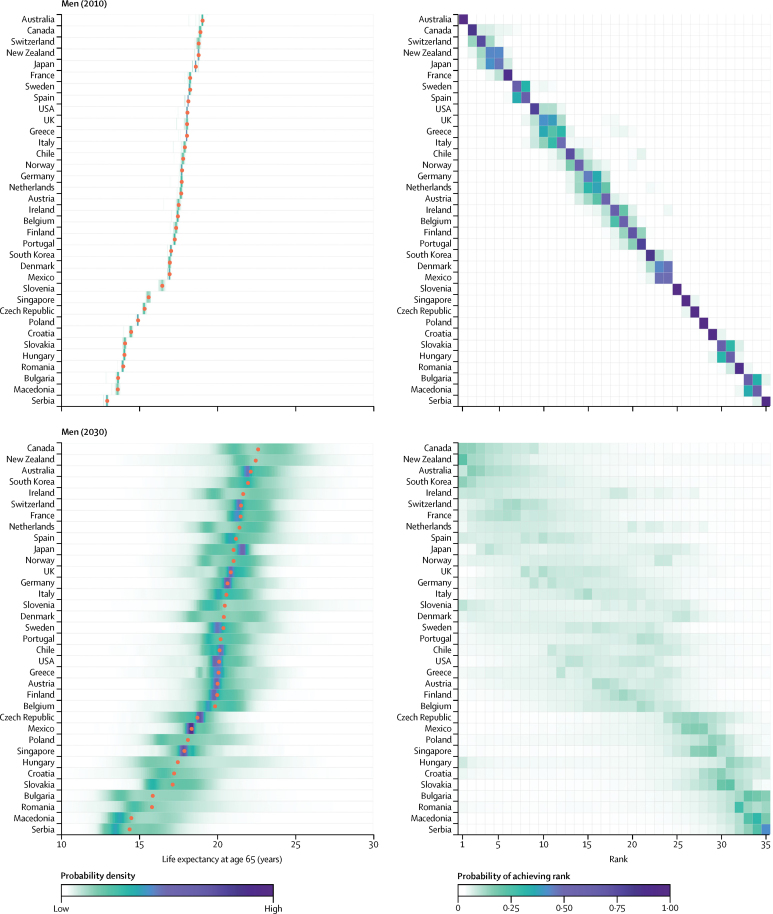
Men's life expectancy at age 65 years in 2010 and 2030 (Left) Posterior distribution of life expectancy at age 65 years and its median value. Red dots show the posterior medians.(Right) Probability distribution for the country's rank. See the appendix (p 37) for numerical values.

**Figure 8:**
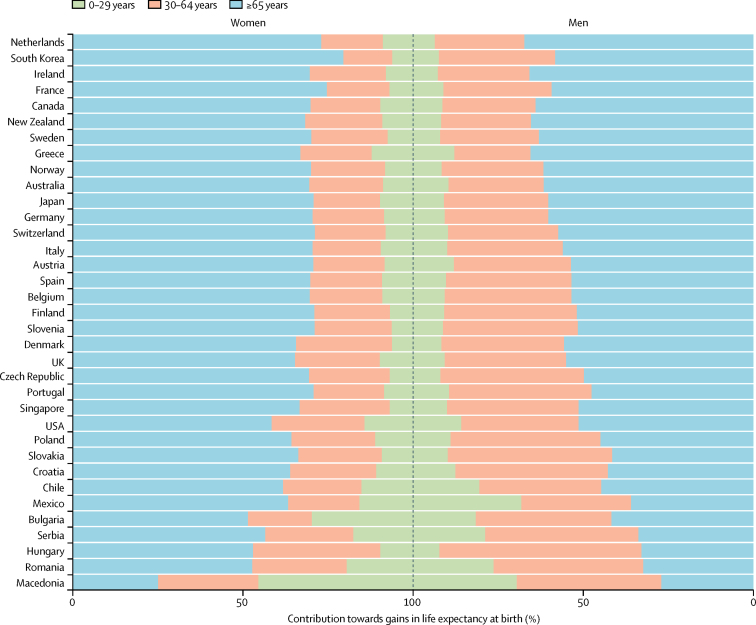
Contributions of mortality decline in three age groups (0–29 years, 30–64 years, and 65 years and older) towards the projected increase in life expectancy at birth

In 2010, life expectancy at age 65 years was highest in Japanese women, at 24·3 years (95% credible interval 23·6–24·7) followed by French women at 23·0 years (22·7–23·2; figure 6). By 2030, 65-year-old women in 19 of the countries analysed here are likely (probability >50%) to have life expectancies greater than 23 years; in 11 of these countries life expectancy at age 65 years is likely to surpass 24 years. Male life expectancy at age 65 years is projected to surpass 20 years—a level that has not been achieved so far—by 2030 in 22 countries with probabilities of over 50% (figure 7). Further reductions are projected in premature mortality, measured by the probability of dying before age 70 years (appendix, pp 28–30). In women, more than half—and in many countries more than two-thirds—of the projected gains in life expectancy at birth will be due to enhanced longevity above age 65 years (figure 8). A similar pattern is also projected among men in Australasia, Asia-Pacific, North America, and western Europe. By contrast, the projected increase in life expectancy at birth for men in central European and Latin American countries will benefit more evenly from gains in the middle ages (age 30–64 years) and older ages.

## Discussion

Using the performance-weighted average of an ensemble of 21 models, we project that there is a high probability that life expectancy will continue to increase in industrialised countries in the Americas, Australasia, central Europe, western Europe, and Asia-Pacific. The increase in life expectancy will be largest in South Korea, some western European countries, and some emerging economies, and smallest in the USA, Japan, Sweden, Greece, Macedonia, and Serbia. Most of the projected gains in life expectancy will occur in older ages, especially in women, furthering the ageing trends in the industrialised world.

Our projections of life expectancy at birth are broadly similar to those made by the UN population division.^11^ The largest differences are for women in Singapore, for whom our projections are lower than those by the UN, and men in Hungary where the reverse occurs. In 2016, Parr and colleagues^12^ projected life expectancy for 14 countries, with substantially higher projections for Japan than ours. The highest projected life expectancy by the UN^11^ (for women in Hong Kong) and Mathers and Loncar^13^ (for women in Japan) is below 90 years; however, our study and that of Parr and colleagues^12^ projected frontiers beyond 90 years (for South Korean women in our work and Japanese women by Parr and colleagues). The projected closing of the female–male gap in life expectancy in most countries in our study is consistent with historical data, when there was no female advantage.^14^ The current gender differences in life expectancy are due to differences in deaths from external causes (injuries) and from conditions such as lung cancer and cardiovascular diseases, whose risk factors (eg, smoking) have different trends in men and women.^14^, ^15^

A key strength of our study is the innovative method for combining projections from multiple models to more completely capture the uncertainty about future trends in mortality and life expectancy. On average, 36% of the variance of final projections for women and 41% for men were due to between-model variance, indicating that the BMA approach is effective in capturing the uncertainty in model choice (see appendix p 40 for the proportion in each country). In addition, BMA projections had a smaller projection error on average than the best model, which improved the validity of the projections.

The key limitation of our work, shared by all projections of the future, is the inability to account for completely unexpected events and changes in the social, technological, and health systems determinants of health. Alternative futures can be considered by envisioning scenarios of such determinants, and analysing how the projected death rates would be modified in such scenarios, as has been done for preventable risk factors.^15^ The model averaging method is inherently reliant on the choice of models included in the ensemble. We tested the performance of the BMA projections when subgroups of the ensemble of models were excluded but found that projection error increased as a result of doing so.

We applied the BMA approach to data from 35 industrialised countries with reliable death registration, for which past trends in mortality were measured with little error, and could be used to fit the projection models. Future work should extend the BMA approach to project mortality for all countries, for many of which past mortality is itself estimated using demographic and statistical models. The simplest option for doing so is to treat the model-estimated past trends as actual measurement, and apply our approach to the resultant death rates. A more complete model would incorporate the uncertainty of estimated past trends (eg, as measurement error), so that this uncertainty can be propagated into the projections. Some previous works have used macroeconomic factors such as per capita national income and risk factors such as smoking in the projections model,^13^ on the premise that such variables might improve projections. Although the associations of these economic and epidemiological variables with mortality can be estimated using historical data, using them to project future mortality requires assumptions about the futures of these factors themselves, or additional models to project them. An alternative approach to using risk factor data is to use an epidemiological model to estimate how much risk factor scenarios modify baseline projections such as those made here.^15^ Finally, we did not project cause-specific mortality. Despite their value for public health and health service planning, whether calculating all-cause mortality projections directly or as the sum of cause-specific projections produces better all-cause mortality forecasts depends on the quality of cause-specific predictions and the relationships between the causes (eg, whether they change in a correlated manner).

Early life expectancy gains in South Korea, which has the highest projected life expectancy, and previous to that in Japan, were driven by declines in deaths from infections in children and adults; more recent gains have been largely due to postponement of death from chronic diseases.^16^, ^17^ These gains were due to broad-based inclusive improvements in economic status^18^ and social capital (including education) in both countries,^19^ which improved childhood and adolescent nutrition (eg, as seen by South Korea and Japan having achieved some of the largest gains in adult height over the past century),^20^ expanded access to primary and secondary health care, and facilitated rapid scale-up of new medical technologies.^16^ South Korea has also maintained lower body-mass index and blood pressure than most western countries,^21^, ^22^ and lower smoking in women. Finally, South Korea and Japan might have lower health inequalities (eg, for cancer and cardiovascular disease mortality, and for self-reported health status) than some of their western counterparts, especially for women.^23^, ^24^, ^25^ Other countries with high projected life expectancy are benefiting from one or more major public health and health-care successes. Examples include high-quality health care that improves prevention and prognosis of cardiovascular diseases and cancers, very low infant mortality, low rates of road traffic injuries and smoking (eg, Australia, Canada, and New Zealand), and low body-mass index (eg, French and Swiss women) and blood pressure (eg, Canada and Australia).^21^, ^22^

By contrast, projected life expectancy is lower in countries with higher levels of young adult mortality and major chronic disease risk factors, and possibly less effective health systems. These countries also tend to have higher social inequalities, which might lower national life expectancy by affecting the entire population or through the poor health of the worst-off social groups and communities, which in turn affects the national average.^26^, ^27^ Notable among poor-performing countries is the USA, whose life expectancy at birth is already lower than most other high-income countries, and is projected to fall further behind such that its 2030 life expectancy at birth might be similar to the Czech Republic for men, and Croatia and Mexico for women. The USA has the highest child and maternal mortality, homicide rate, and body-mass index of any high-income country, and was the first of high-income countries to experience a halt or possibly reversal of increase in height in adulthood, which is associated with higher longevity.^20^, ^21^, ^28^, ^29^, ^30^ The USA is also the only country in the OECD without universal health coverage, and has the largest share of unmet health-care needs due to financial costs.^25^ Not only does the USA have high and rising health inequalities, but also life expectancy has stagnated or even declined in some population subgroups.^1^, ^2^ Therefore, the poor recent and projected US performance is at least partly due to high and inequitable mortality from chronic diseases and violence, and insufficient and inequitable health care.

With the exception of obesity, effective strategies and policies exist to modify the important behavioural and health systems determinants of adult mortality.^31^ The cornerstone of these strategies is an equitable and effective health system that provides universal free access to high-quality primary and secondary care for prevention and treatment, and uses regulation and economic tools (eg, taxes) for substantially reducing tobacco and harmful alcohol use. Our projected longevity gains, and the contributions of older age longevity to these gains, also have major implications for health and social services.^5^ There is a need for health services that provide long-term care for the increasing number of older people who are affected by multi-morbidity and limited mobility.^32^ The health-care needs go beyond simply increasing the number of facilities and personnel, itself a challenge in the current era of austerity, and should involve considerations about how and where care is delivered including more integrated care in the community setting or even at home. At the same time, healthy ageing through the life course can prevent or delay the chronic conditions that affect older people; assistive technologies that compensate for loss of sensory and motor abilities, and appropriate changes to the built environment and transportation services can support independent living and mobility.^32^ Furthermore, social security and pensions will face additional payouts with extended longevity, which will further stress them unless work and retirement practices change, for example through gradual transition to retirement or later retirement age.^5^ Although rising life expectancy necessitates policies that can support healthy ageing, reframing of education–work–retirement practices, and investment in health and social care, our projections show the continued success of extending longevity.

## Figures and Tables

**Table:** Mortality data availability by country

	**Start year**	**End year**	**Missing data years**
Australia*	1960	2012	..
Austria*	1960	2014	..
Belgium*	1960	2012	..
Bulgaria	1964	2012	..
Canada*	1960	2011	..
Chile*	1960	2013	..
Croatia	1982	2013	..
Czech Republic	1982	2013	..
Denmark*	1960	2012	..
Finland*	1960	2013	..
France*	1960	2012	..
Germany†	1973	2013	1975, 1979
Greece	1961	2012	..
Hungary*	1960	2013	1969–74
Ireland*	1960	2012	..
Italy*	1960	2012	..
Japan*	1960	2013	..
Macedonia	1982	2010	..
Mexico*	1960	2013	..
Netherlands*	1960	2013	..
New Zealand*	1960	2011	..
Norway*	1960	2013	..
Poland*	1960	2013	..
Portugal*	1960	2013	1969, 1971–73, 1975–79
Romania*	1960	2012	1979
Serbia	1985	2013	..
Singapore	1963	2014	..
Slovakia	1982	2014	..
Slovenia	1982	2010	..
South Korea‡	1985	2014	..
Spain*	1960	2013	1975
Sweden*	1960	2014	..
Switzerland*	1960	2012	..
UK*	1960	2013	..
USA*	1960	2013	..

* Used for measuring the performance of the Bayesian model averaging projections.

† For pre-unification years, we added German Democratic Republic and Federal Republic of Germany death counts and populations.

‡ Data for South Korea in 2014 were taken directly from the South Korean Statistical Information Service database.
