# Supplementary material for: Future life expectancy in 35 industrialised countries: projections with a Bayesian model ensemble
Source: Lancet. 2017 Apr 1;389(10076):1323–35. doi: 10.1016/S0140-6736(16)32381-9 (PMC5387671; doi:10.1016/S0140-6736(16)32381-9)
Supplement: Supplementary appendix [file mmc1.pdf]

THE LANCET

Supplementary appendix

This appendix formed part of the original submission and has been peer reviewed. We post it as supplied by the authors.

Supplement to: Kontis V, Bennett JE, Mathers CD, Li G, Foreman K, Ezzati M. Future life expectancy in 35 industrialised countries: projections with a Bayesian model ensemble. *Lancet* 2017; published online Feb 21. http://dx.doi.org/10.1016/S0140-6736(16)32381-9.

Webappendix

Table of contents

Appendix Text 1: Data 3

Appendix Text 2: Model ensemble 4

Appendix Text 3: Calculating life expectancy 7

Appendix Text 4: Measuring the performance of Bayesian model average projections 8

Appendix Text 5: Metric used to measure projection error 10

References 11

Appendix Figure 1: Absolute projection bias (A) and ranking of absolute projection bias (B) for the 21 models in the ensemble (numbered from 1 to 21 as shown in Appendix Table 5) and 35 analysis countries. 13

Appendix Figure 2: Model weights for the 21 models in the ensemble (numbered from 1 to 21 as shown in Appendix Table 5) and 35 analysis countries. 16

Appendix Figure 3: Projection bias in life expectancy at birth by length of validation period. 18

Appendix Figure 4: Trends in life expectancy at birth. 20

Appendix Figure 5: Women's (A) and men's (B) probability of dying before 70 years of age in 2010 and 2030. 28

Appendix Figure 6: Comparison of projection bias in life expectancy at birth vs projection bias in log-transformed death rates. 31

Appendix Figure 7: Comparison of projection deviation vs projection bias for life expectancy. 33

Appendix Table 1: Ninety percent coverage of life expectancy projections in validation analyses. 35

Appendix Table 2: Life expectancy at birth in 2010 and 2030 36

Appendix Table 3: Life expectancy at age 65 years 37

Appendix Table 4: Probability of dying before 70 years of age 38

Appendix Table 5: Numbered list of ensemble models. 39

Appendix Table 6: Total variance of the projected life expectancy in 2030 and the proportion of total variance that is due to between-model variance, by country and sex. 40

Appendix Text 1: Data

Pre-processing steps

The WHO uses demographic methods to assess the completeness of death registration data for each country.[1] Ninety-six percent of country-year-sex combinations in our analysis had a completeness of 90% or higher, with the remaining ones having completeness between 70% and 90%. We adjusted the number of deaths by the extent of under-registration (which is the inverse of completeness) for each country, year and sex.

Five countries had some missing data: Romania and Spain had one year of missing data, Germany two, Hungary six and Portugal nine (Table in main paper). As a pre-processing step, we imputed the missing years by linear interpolation between the death rates of adjacent years.

Some researchers have found that smoothing death rates before fitting models reduces the projection error.[2, 3] Therefore, we investigated the effect of smoothing death rates before fitting our models. We tested the following approaches to smoothing:

- Smoothing log-transformed death rates using a simple unweighted 5-year moving average.
- Smoothing log-transformed death rates using a 5-year moving average, giving the central year of the average three times the weight of the outermost years, and the two years adjacent to the centre two times the weight of the outermost years.
- No smoothing.

We found that the projections using unweighted smoothing had the smallest projection error (calculated as described in Appendix Text 4). Therefore, we used this approach to smooth the data before model fitting.

Appendix Text 2: Model ensemble

As described in the main text, we developed an ensemble of 21 models for projecting age-specific death rates, which were in turn used to calculate projected life expectancy. These models were formulated to incorporate established features of death rates in relation to age and birth cohort, and over time, as well as statistical considerations such as the extent of smoothing over age and birth cohort, and how much weight to give older data points compared to more recent ones. Specifically, for a given country and sex, the number of deaths in age group a and year t follows a Poisson distribution

$$\text{Deaths}_{at} \sim \text{Poisson}(m_{at} \cdot \text{Population}_{at}) ,$$

where m_{at} is the death rate. In all of our models, log-transformed death rates were modelled as a sum of components that may depend on time, age and cohort, as described below. See Appendix Table 5 for a summary table of models in the ensemble.

Age-time models

These models contain terms to capture the overall level and rate of change of mortality, as well as age-specific offsets that allow each age group's mortality level and trend to deviate from the common terms. Log-transformed death rates are modelled as

$$\log(m_{at}) = \theta_0 + \theta_a + \beta_0 t + \beta_a t + \nu_{at} + \varepsilon_{at} ,$$

where θ_0 is the common intercept for death rates across age groups; β_0 quantifies the common trend across ages. θ_a and β_a measure deviation from the common level and trend, respectively, by age group.

The choice of prior on these age-specific terms determines the level of borrowing of strength and smoothing over age. We defined two versions of this model: one with an independent and identically-distributed (IID) structure $\theta_a \overset{\text{IID}}{\sim} \mathcal{N}\left(0, \sigma^2_{\theta_a}\right)$, $\beta_a \overset{\text{IID}}{\sim} \mathcal{N}\left(0, \sigma^2_{\beta_a}\right)$ and one with a first-order random walk structure $\theta_a \sim \mathcal{N}\left(\theta_{a-1}, \sigma^2_{\theta_a}\right), \beta_a \sim \mathcal{N}\left(\beta_{a-1}, \sigma^2_{\beta_a}\right)$. The first-order random walk allows borrowing of strength across adjacent age groups and typically results in greater smoothness across the age groups.

For each age a, the term ν_{at} is a first-order random walk over time that allows for nonlinearity in the age trends, beyond what is accounted for by other trend components. The degree of correlation among these age-specific random walks is modelled via a parameter ρ that is estimated by the model, as detailed in a methodological reference.[4] The ε_{at}, modelled as $\mathcal{N}(0, \sigma^2_\varepsilon)$, account for additional variability in the data not captured by other components in the models.

Age-time-cohort models

We extended the age-time models by including an additional cohort component on trend which allows trends in death rates to vary by birth cohort. We did not include a cohort

intercept because including components for age, time and birth cohort on mortality level makes the estimation non-identifiable.[5, 6] Log-transformed death rates were modelled as

$$\log(m_{at}) = \theta_0 + \theta_a + \beta_0 t + \beta_a t + \beta_c t + \nu_{at} + \varepsilon_{at}\,.$$

The cohort-specific slopes β_c were assigned a first-order random walk prior, $\beta_c \sim \mathcal{N}\left(\beta_{c-1}, \sigma^2_{\beta_c}\right)$, allowing trends in adjacent birth cohorts to be more similar than in those born in different eras. The remaining terms were the same as in the age-time models above.

Piecewise-linear age-time and age-time-cohort models

Death rates in more recent years may be better predictors of future mortality trends than those in earlier years, which may nonetheless be informative about levels of mortality in relation to age or country. We implemented models to consider this possibility, using a piecewise linear trend component. Specifically, we added a knot at time K to the trend term, and estimated the slopes as two segments that connect at this knot. In the model specification above, instead of a single parameter for the common slope β_0, we used two parameters, $\beta_0^{(1)}$ and $\beta_0^{(2)}$, and replaced the term $\beta_0 t$ in the equation for $\log(m_{at})$ by

$$\begin{cases} \beta_0^{(1)} t, & \text{if } t \leq K, \\ \beta_0^{(1)} K + \beta_0^{(2)} (t - K), & \text{if } t > K. \end{cases}$$

We also replaced the age and cohort slopes with similar piecewise linear terms. When projecting death rates, it is the second segment of the slope $\beta_0^{(2)}$ that determines the overall change of mortality over time. For each age-time model and each age-time-cohort model, we used two piecewise linear versions, one placing the knot 6 years and the other 10 years before the end of the data respectively. These two knot placements were chosen so that the two models were as distinct as possible, while still allowing for enough data on both sides of the knot to estimate all the parameters.

Weighted likelihood age-time and age-time-cohort models

A second way to allow more influence from more recent data than earlier ones is to multiply the likelihood function by a time-dependent decay function.[7] These weights decay exponentially in time, as a function of a positive parameter λ which determines the rate of decay. Specifically, data n years before the end are assigned weight $(1-\lambda)^n$ (normalised so that weights sum to 1), so that more recent data receive the largest weight and early data receive little weight. We used a λ equal to 0.05, which is small enough to distinguish these models from the unweighted ones, and large enough to still let the older data have some impact on the model parameters. We defined a weighted-likelihood version of each age-time model and each age-time-cohort model.

Hyper-priors

We used weakly informative priors so that parameter estimation was driven by the data.[8] The hyper-priors were defined on the logarithm of the precisions of the random effects, for example on $\log(1/\sigma^2_\theta)$. These were modelled as $\text{logGamma}(\alpha, \beta)$ distributions with

shape $\alpha = 1$ and rate $\beta = 0.001$. The same hyper-priors were used for all precision parameters of the random effects in all models described above. For the common slopes and intercepts, we used $\mathcal{N}(0, 1000)$.

Lee-Carter models

A popular model for mortality forecasting is the one introduced by Lee and Carter, used initially in the USA.[9] In this model, the logarithm of death rates is estimated as

$$\log(m_{at}) = \theta_a + \beta_a \gamma_t + \varepsilon_{at} \, ,$$

where θ_a reflects average age-specific death rates over time, β_a captures variation of mortality across age and γ_t captures variation across time, while ε_{at} is a zero mean term that accounts for additional variability. The $\beta_a \gamma_t$ term is calculated using principal component decomposition, together with a first-order random walk over time.[9, 10] We also implemented extensions that used multiple principal components, $\beta_a^{(1)} \gamma_t^{(1)} + \cdots + \beta_a^{(k)} \gamma_t^{(k)}$, which are able to capture more information across age and time. We included in our family of models the original Lee Carter model as well as extensions with up to 5 principal components ($k = 1, \ldots 5$).

Implementation

All models were fitted using the R software (version 3.2.5).[11] The Lee-Carter models were fitted using the methods outlined in Girosi and King,[10] and their projection distributions were obtained by sampling draws using the estimated mean and variance from the model. All other models were fitted using integrated nested Laplace approximation,[12] implemented in the R-INLA software (version 0.0-1455098891).[13] We took a number of draws from the posterior distribution of age-specific death rates under each model proportional to the weight of that model, and pooled the draws to obtain the posterior distribution of age-specific death rates under the BMA. These projected death rates were then used to give the posterior distributions of the projection under the BMA of life expectancy at birth and at aged 65 and the probability of dying under age 70.

Appendix Text 3: Calculating life expectancy

We used deaths and population in 5-year age groups from 0-4 years to 80-84 years and 85 years and older. We calculated life expectancy using age-specific death rates by applying life table methods.[14, 15] Life expectancy calculations need an estimate of the average number of years lived by those who die in each five-year age group as well as those who are 85 years of age and older. For the youngest age group (0-4 years), we used UN life tables (from WPP 2012) and projected the last available year forward as a flat line to 2030. In practice, since the countries in our analysis have low child mortality, our life expectancy estimates are not sensitive to changes and variations in the number of years lived by those who die in the youngest age group. For the average number of years lived by those who die in the 5-year age groups 5-9 to 80-84 we used the methods presented by Preston et al. (Chapter 3).[14] We used the Kannisto-Thatcher method to expand the terminal (85 years and older) age group of the life table.[16] This method is designed for use in low-mortality populations and is used by the UN Population Division, the WHO,[15] and the Human Mortality Database.[17]

Appendix Text 4: Measuring the performance of Bayesian model average projections

To measure the performance of the model average, we withheld 22 recent years of data. We withheld a significant portion of the data to validate our approach in a test that is analogous to its actual task of medium-term projection, and is also supported by findings in other studies.[2, 18] We used the remaining data to produce BMA projections for this withheld period, as described in the methods section of the main paper, and examined how well the BMA projections reproduce the withheld data. We used the 24 countries with data over the 1960-2010 period (Table in main paper) for this test, because their long time series of data allowed withholding some years and yet having sufficient data for generating BMA projections. The steps for measuring the performance of BMA projections are described below and the results are provided in Appendix Figure 3 and Appendix Table 1.

Step A1: Measuring the bias of projections from individual models
We used the first 16 years of data (1960-1975) to estimate model parameters, which we then used to project for 1976-1988 (i.e., 13 years, the same period length as used to calculate model weights when producing the final projections as described in the main paper), and measured the projection bias of individual models as described in the main paper.

Step A2: Calculating model weights
We used the projection bias in life expectancy over the withheld data in Step A1 to calculate model weights as described in the main paper.

Step A3: Calculating the BMA projections
We used the data for 1960-1988 to estimate model parameters, and used them to project for 1989-2010. We calculated the BMA projections over 1989-2010 using the model weights from Step A2.

Step A4: Evaluating the performance of BMA projections
We compared the BMA projections with the withheld data to calculate the projection bias. In addition, we calculated the projection bias of the model with the smallest projection bias in Step A1 over the same period (1989-2010) (that is, the model with largest weight) to see how the BMA projection performs relative to the best model for each country and sex. The average (across all 24 countries in our performance testing analysis) BMA projection bias was smaller than that of the best model for both sexes (Appendix Figure 3).

We also tested if the BMA, as well as the best model, performed differentially better or worse for shorter vs. longer term projections. To do so, we repeated the above steps allowing the withheld data length to vary from 1 year (2010) to 22 years (1989-2010), coinciding with shorter vs. longer projection periods. We determined the best model for each combination of country, sex and validation period. As the length of the validation period increased, the relative advantage of the BMA compared to the best model increased (Appendix Figure 3). This progressively improved performance of the BMA

likely occurs because death rates in any country have trends whose properties vary over time, and hence in the longer term are better captured by the average of a portfolio of models than by a single model.

Projection data coverage

We also report the 90% coverage of projections, which measures how well the posterior distributions of estimated life expectancies coincide with the observed death rates, for the 1989-2010 validation period. If projected life expectancies and their uncertainties are well estimated, the estimated 90% credible intervals should cover 90% of the withheld data points. Coverage estimates took into account the variations both due to the Bayesian model parameters and due to the stochastic nature of death counts; the latter was dealt with by adding in Poisson variation.[19, 20] Results are shown in Appendix Table 1.

Appendix Text 5: Metric used to measure projection error

We measured projection performance in out-of-sample tests using withheld years of data, as described in Step 1 in the main paper and in Appendix Text 4. Model performance can be measured using bias (sum of the differences between the withheld data and the projected values) or deviation (sum of the absolute value of these differences). Bias and deviation can both be calculated for death rates or for life expectancy at birth. We calculated the projection performance using the bias in life expectancy at birth. The choice was motivated by the fact that life expectancy is our primary outcome, rather than age-specific rates, and that life expectancy at birth is a single quantity that combines the death rates of all age groups in a coherent manner. To investigate the sensitivity of projection errors to our choice of metric, we compared bias in life expectancy at birth to (i) bias in the logarithm of death rates and (ii) deviation in life expectancy at birth.

Comparing projection bias in life expectancy to projection bias in death rates

We calculated projection bias for both life expectancy and age-specific death rates by subtracting projections from withheld data. Projection bias in death rates was calculated by averaging projection bias in logarithm of age-specific death rates across age groups. We used the logarithm of death rates in order to account for the large variation of death rates across age groups, and avoid the oldest age groups (with orders of magnitude higher mortality than the younger ones) dominating the projection error. The comparison between bias in life expectancy and bias in log-transformed death rates showed that these two metrics are highly correlated (Appendix Figure 6).

Comparing projection bias to projection deviation

We calculated projection deviation in life expectancy using the absolute differences between projected and withheld data. Comparing projection bias to projection deviation in life expectancy showed that these metrics are highly correlated (Appendix Figure 7). In other words, model projections tend to lie either above the test data or below the test data for all years, rather than fluctuate through it. Therefore, the model weights would be similar using either metric.

References

1. World Health Organization. WHO methods and data sources for country-level causes of death 2000-2012. 2014. http://www.who.int/healthinfo/statistics/GlobalCOD_method.pdf.
2. Hyndman RJ, Ullah MS. Robust forecasting of mortality and fertility rates: A functional data approach. *Computational Statistics & Data Analysis* 2007; **51**(10): 4942-56.
3. Shang HL, Booth H, Hyndman RJ. Point and interval forecasts of mortality rates and life expectancy: A comparison of ten principal component methods. *Demogr Res* 2011; **25**: 173-214.
4. Riebler A, Held L, Rue H. Estimation and extrapolation of time trends in registry data - Borrowing strength from related populations. *Ann Appl Stat* 2012; **6**(1): 304-33.
5. Clayton D, Schifflers E. Models for temporal variation in cancer rates. II: Age-period-cohort models. *Statistics in medicine* 1987; **6**(4): 469-81.
6. Holford TR. The estimation of age, period and cohort effects for vital rates. *Biometrics* 1983; **39**(2): 311-24.
7. Hyndman RJ, Booth H, Yasmeen F. Coherent mortality forecasting: the product-ratio method with functional time series models. *Demography* 2013; **50**(1): 261-83.
8. Gelman A, Carlin JB, Stern HS, Dunson DB, Vehtari A, Rubin DB. Bayesian data analysis, Third Edition. Boca Raton: Chapman and Hall/CRC 2013.
9. Lee RD, Carter LR. Modeling and forecasting U.S. mortality. *J Am Stat Assoc* 1992; **87**(419): 659-71.
10. Girosi F, King G. Demographic forecasting. Princeton, NJ: Princeton University Press; 2008.
11. R Core Team. R: A language and environment for statistical computing. Vienna, Austria: R Foundation for Statistical Computing; 2015.
12. Rue H, Martino S, Chopin N. Approximate Bayesian inference for latent Gaussian models by using integrated nested Laplace approximations. *J Roy Stat Soc B* 2009; **71**: 319-92.
13. Lindgren F, Rue H. Bayesian spatial modelling with R-INLA. *J Stat Softw* 2015; **63**(19): 1-25.
14. Preston S, Heuveline P, Guillot M. Demography: measuring and modeling population processes: Wiley-Blackwell; 2001.
15. World Health Organization. WHO methods and data sources for life tables 1990-2015. 2016. http://www.who.int/healthinfo/statistics/LT_method.pdf.
16. Thatcher AR, Kannisto V, Vaupel JW. The force of mortality at ages 80 to 120. Odense monographs on population aging. Odense: Odense University Press 1998.
17. Wilmoth JR, Andreev KV, Jdaoy DA, Glei DA. Methods protocol for the human mortality database. 2007. http://www.mortality.org/Public/Docs/MethodsProtocol.pdf.
18. Eklund J, Karlsson S. Forecast combination and model averaging using predictive measures. *Economet Rev* 2007; **26**(2-4): 329-63.
19. Christoffersen PF. Evaluating interval forecasts. *Int Econ Rev* 1998; **39**(4): 841-62.
20. Brillinger DR. The natural variability of vital-rates and associated statistics. *Biometrics* 1986; **42**(4): 693-712.

21. Hoeting JA, Madigan D, Raftery AE, Volinsky CT. Bayesian model averaging: A tutorial. *Stat Sci* 1999; **14**(4): 382-401.
22. Raftery AE, Gneiting T, Balabdaoui F, Polakowski M. Using Bayesian model averaging to calibrate forecast ensembles. *Monthly Weather Review* 2005; **133**(5): 1155-74.

Appendix Figure 1: Absolute projection bias (A) and ranking of absolute projection bias (B) for the 21 models in the ensemble (numbered from 1 to 21 as shown in Appendix Table 5) and 35 analysis countries. The projection bias for each model and country is the difference between the projected life expectancy and withheld data averaged over all 13 years of withheld data.

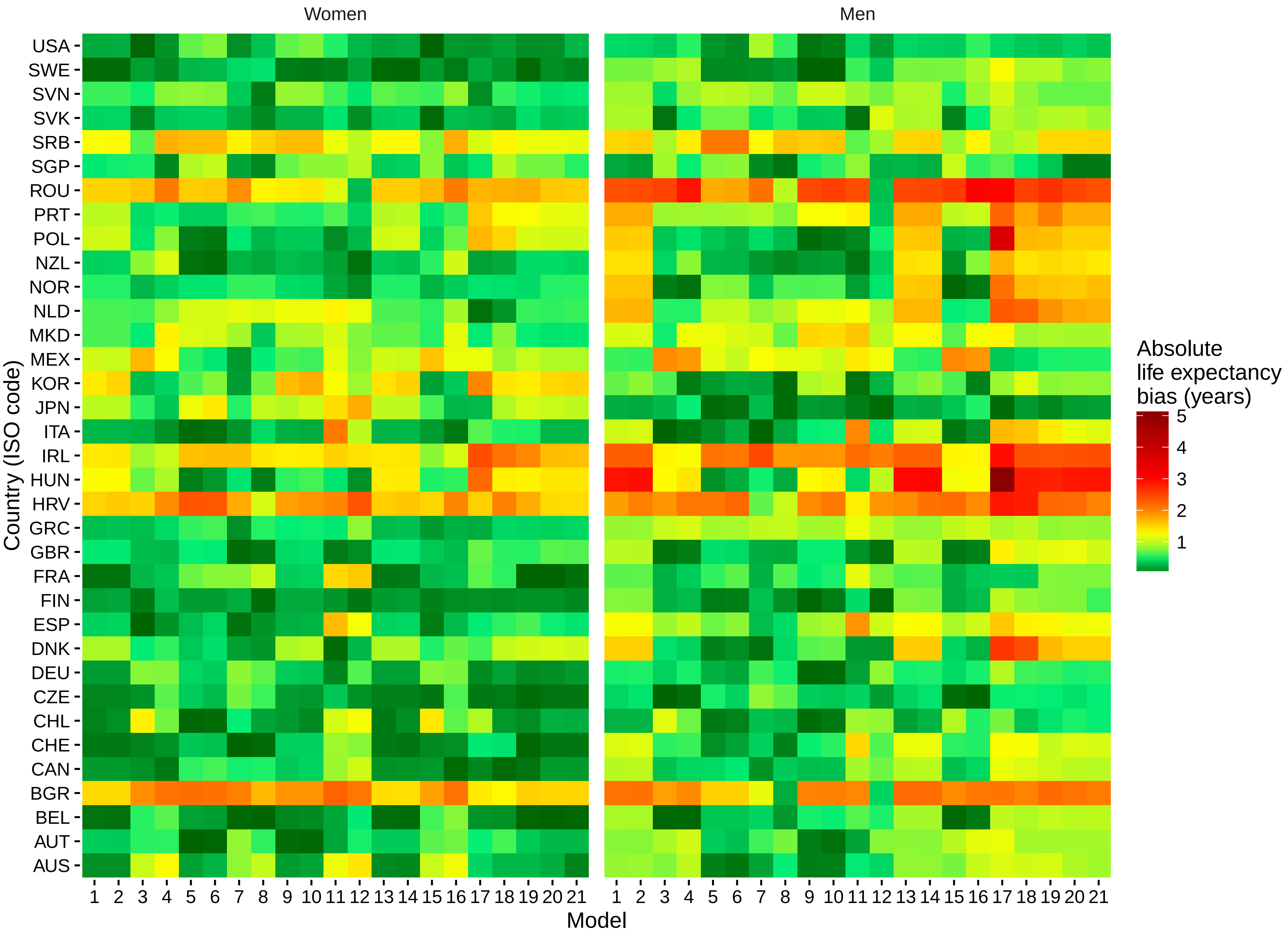
Women
Men
USA
SWE
SVN
SVK
SRB
SGP
ROU
PRT
POL
NZL
NOR
NLD
MKD
MEX
KOR
JPN
ITA
IRL
HUN
HRV
GRC
GBR
FRA
FIN
ESP
DNK
DEU
CZE
CHL
CHE
CAN
BGR
BEL
AUT
AUS
Country (ISO code)
1 2 3 4 5 6 7 8 9 10 11 12 13 14 15 16 17 18 19 20 21
Model
Absolute life expectancy bias (years)
5
4
3
2
1

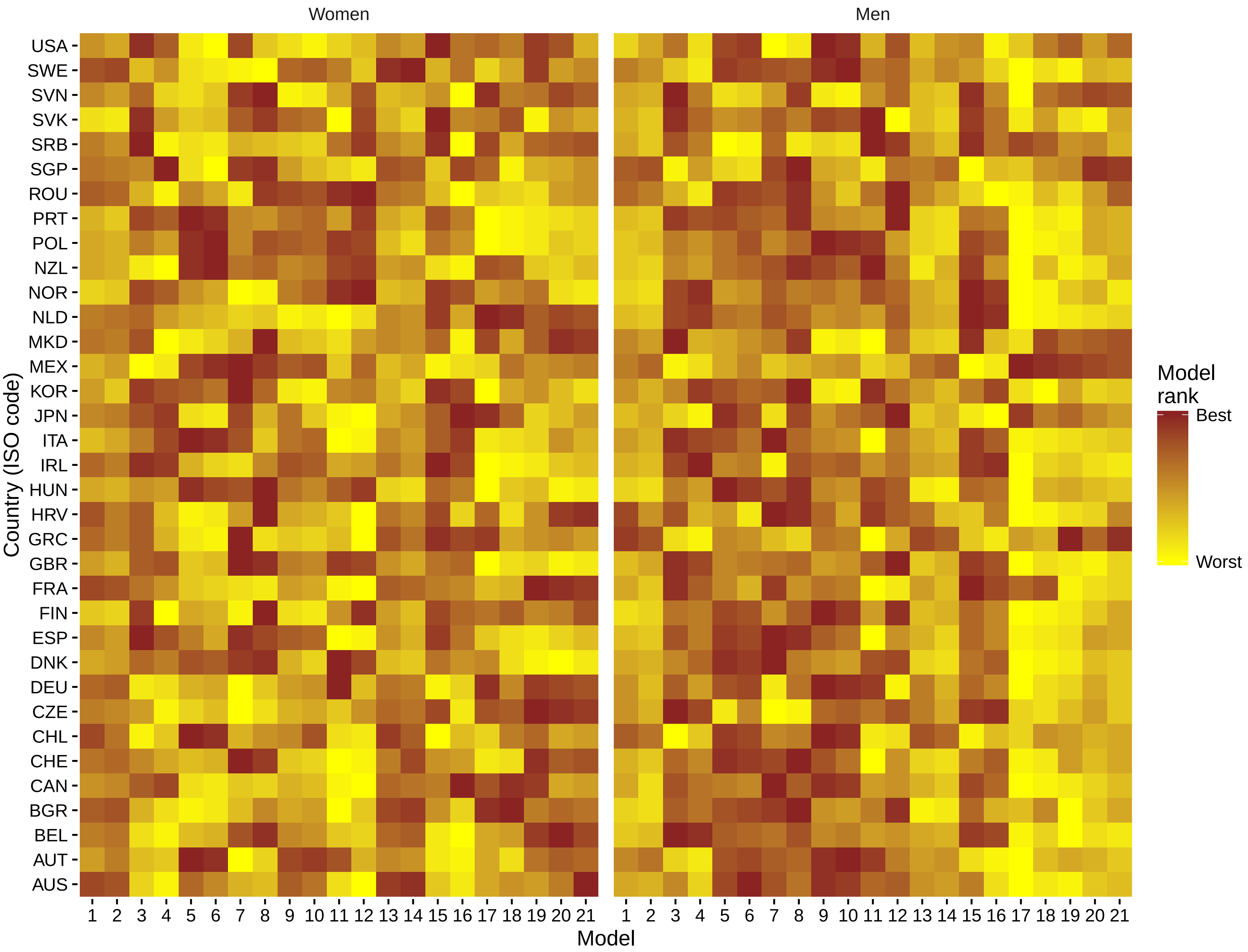

Women
Men
USA
SWE
SVN
SVK
SRB
SGP
ROU
PRT
POL
NZL
NOR
NLD
MKD
MEX
KOR
JPN
ITA
IRL
HUN
HRV
GRC
GBR
FRA
FIN
ESP
DNK
DEU
CZE
CHL
CHE
CAN
BGR
BEL
AUT
AUS
Country (ISO code)
1 2 3 4 5 6 7 8 9 10 11 12 13 14 15 16 17 18 19 20 21
Model
Model rank
Best
Worst

Appendix Figure 2: Model weights for the 21 models in the ensemble (numbered from 1 to 21 as shown in Appendix Table 5) and 35 analysis countries. Each model was assigned a weight of exp(-|Projection bias|); the 21 weights were normalised to sum to 1. The projection bias for each model and country is the difference between the projected life expectancy and withheld data averaged over all 13 years of withheld data.

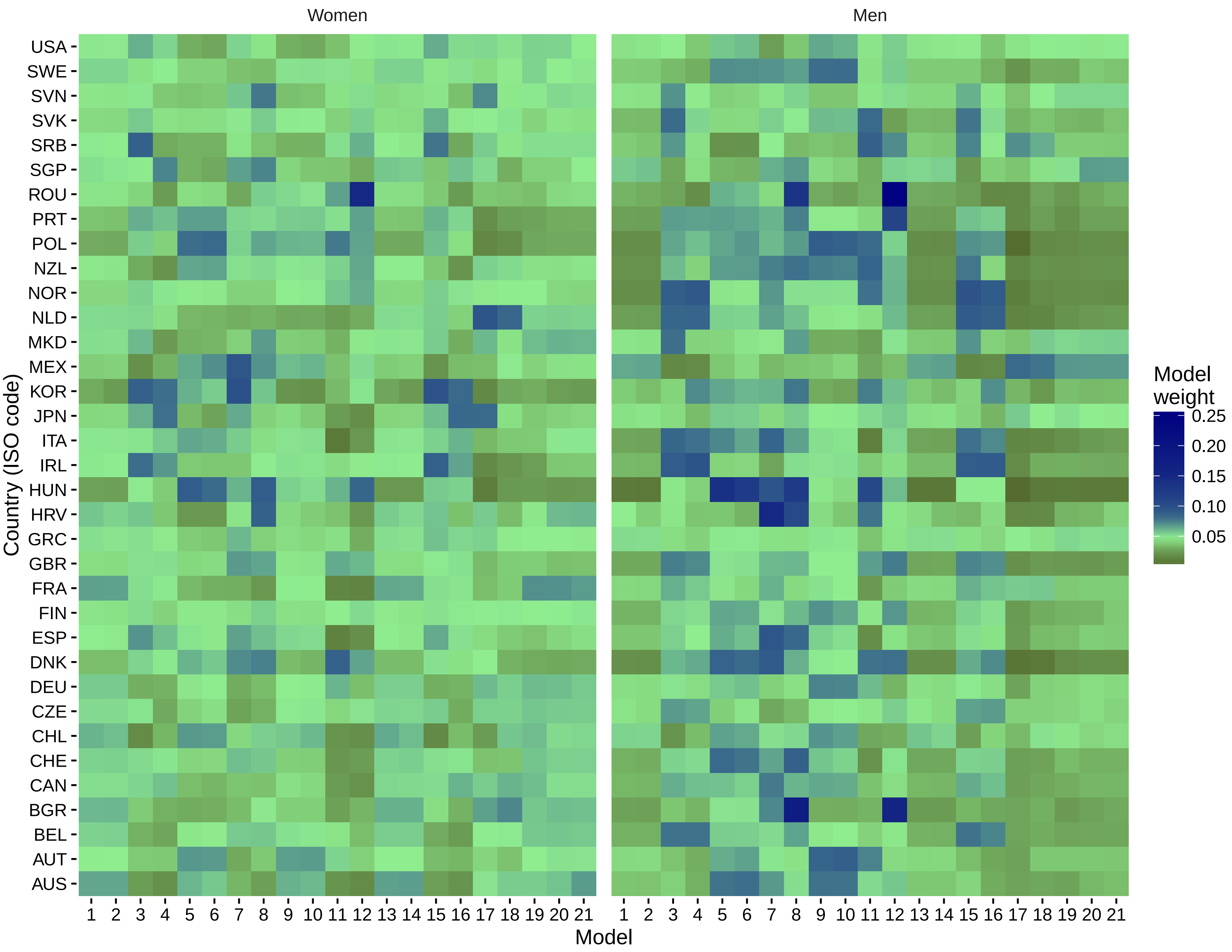
Women
Men
Country (ISO code)
USA
SWE
SVN
SVK
SRB
SGP
ROU
PRT
POL
NZL
NOR
NLD
MKD
MEX
KOR
JPN
ITA
IRL
HUN
HRV
GRC
GBR
FRA
FIN
ESP
DNK
DEU
CZE
CHL
CHE
CAN
BGR
BEL
AUT
AUS
1 2 3 4 5 6 7 8 9 10 11 12 13 14 15 16 17 18 19 20 21
Model
Model weight
0.25
0.20
0.15
0.10
0.05

Appendix Figure 3: Projection bias in life expectancy at birth by length of validation period. The average projection bias from the model average is compared with that resulting from the best model for each country, sex and validation period. The data used in the graph is the average projection bias across all countries for validation periods of 1-22 years.

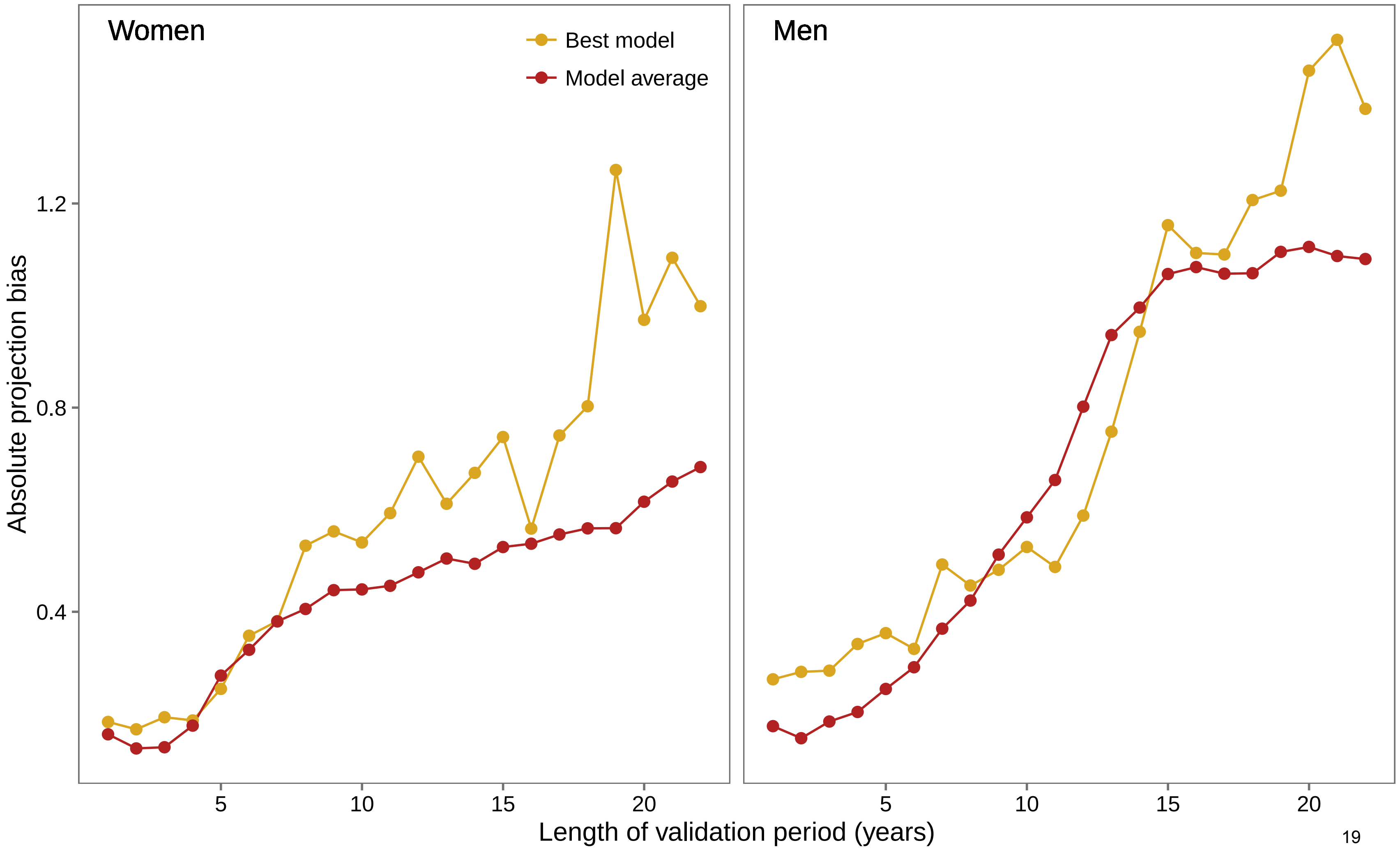
Women
Men
Best model
Model average
Absolute projection bias
1.2
0.8
0.4
5
10
15
20
Length of validation period (years)

Appendix Figure 4: Trends in life expectancy at birth. The black dots indicate observed data (linearly interpolated for the four countries with some missing data as detailed in Appendix Text 1). The grey-shaded areas show levels of credible intervals around the median, from 10% (dark grey) to 95% (light grey).

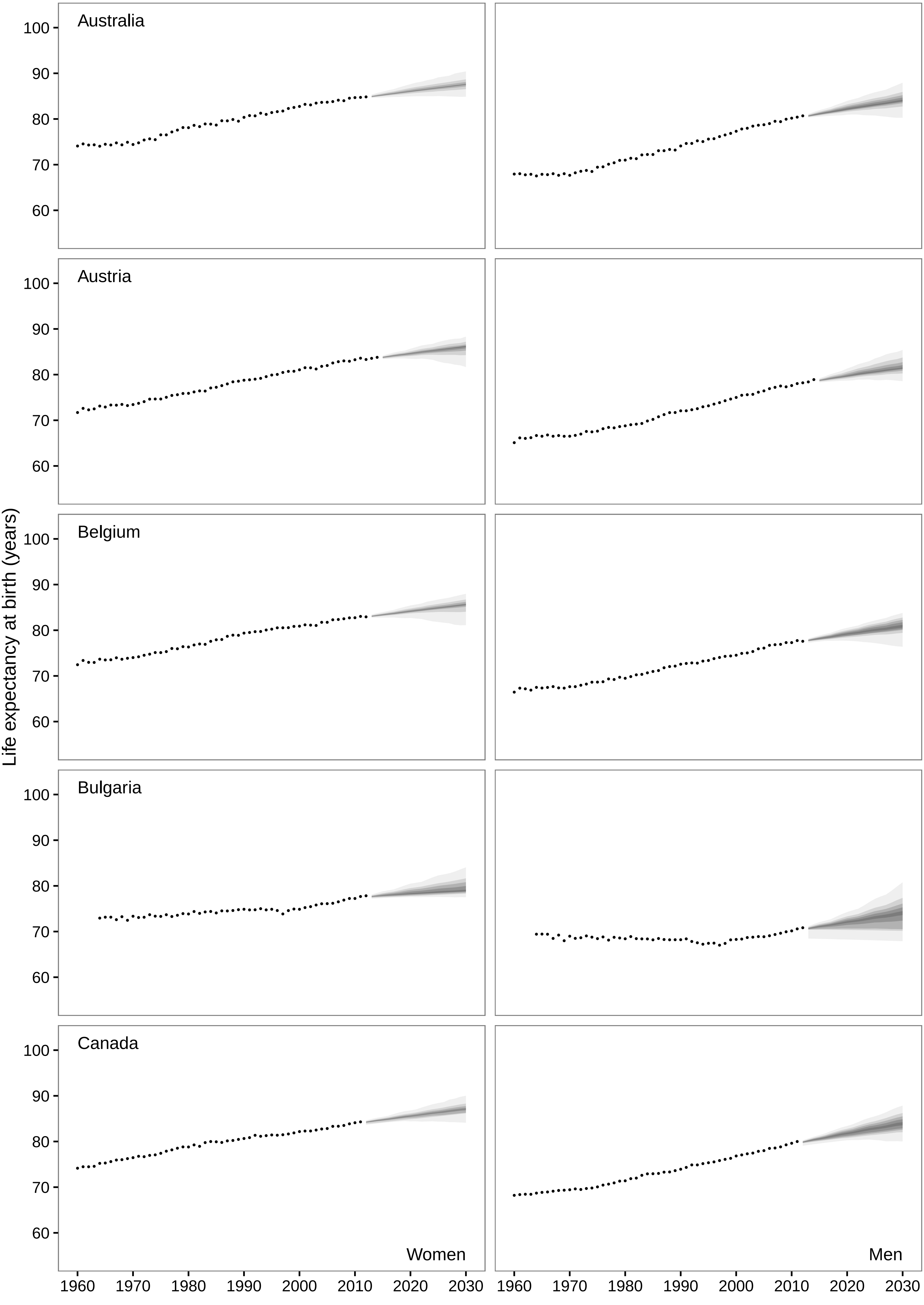

Life expectancy at birth (years)
Australia
Austria
Belgium
Bulgaria
Canada
Women
Men
100
90
80
70
60
1960
1970
1980
1990
2000
2010
2020
2030

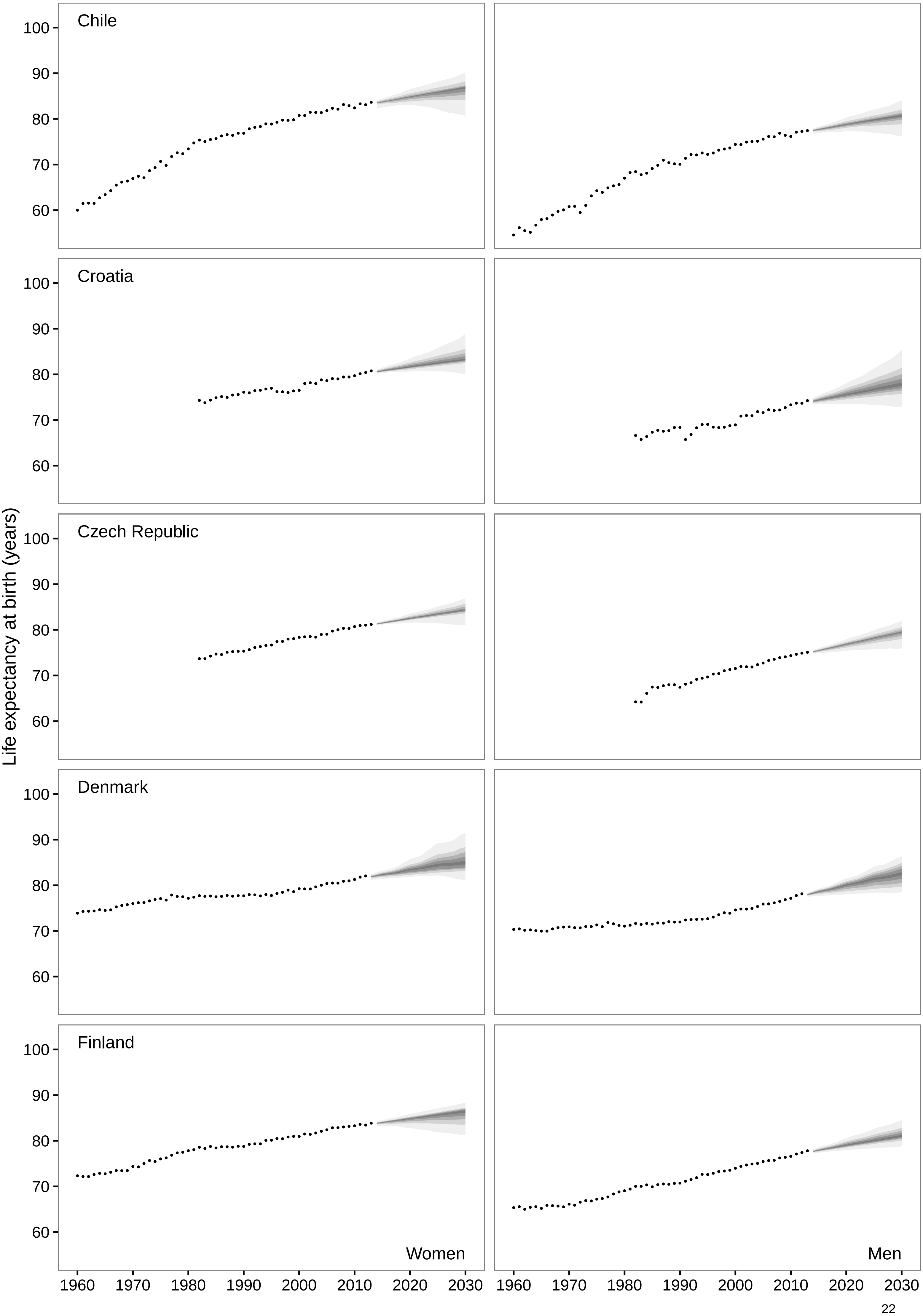

Chile
Croatia
Czech Republic
Denmark
Finland
Life expectancy at birth (years)
100
90
80
70
60
1960
1970
1980
1990
2000
2010
2020
2030
Women
Men

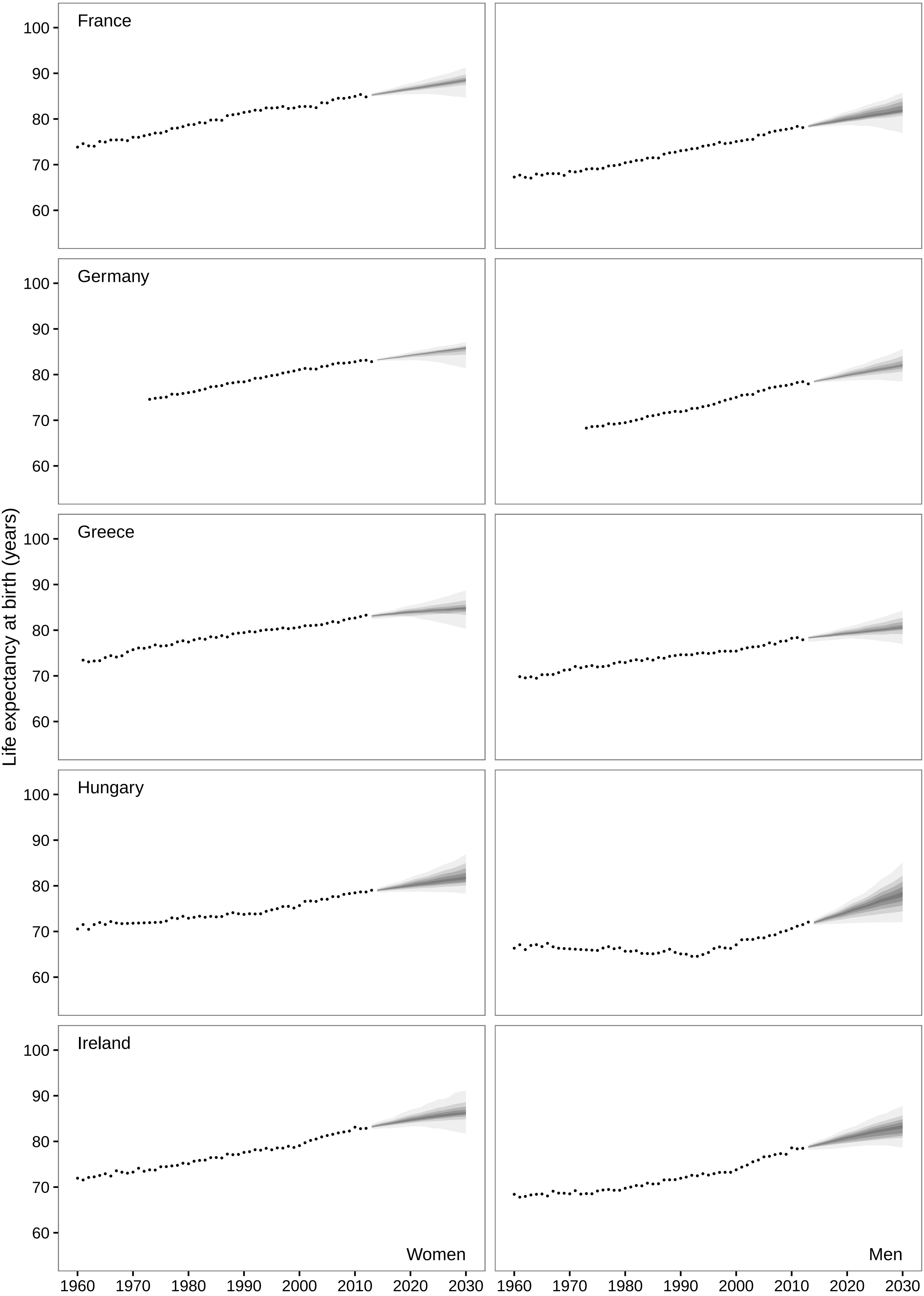
France
Germany
Greece
Hungary
Ireland
Life expectancy at birth (years)
100
90
80
70
60
Women
Men
1960
1970
1980
1990
2000
2010
2020
2030

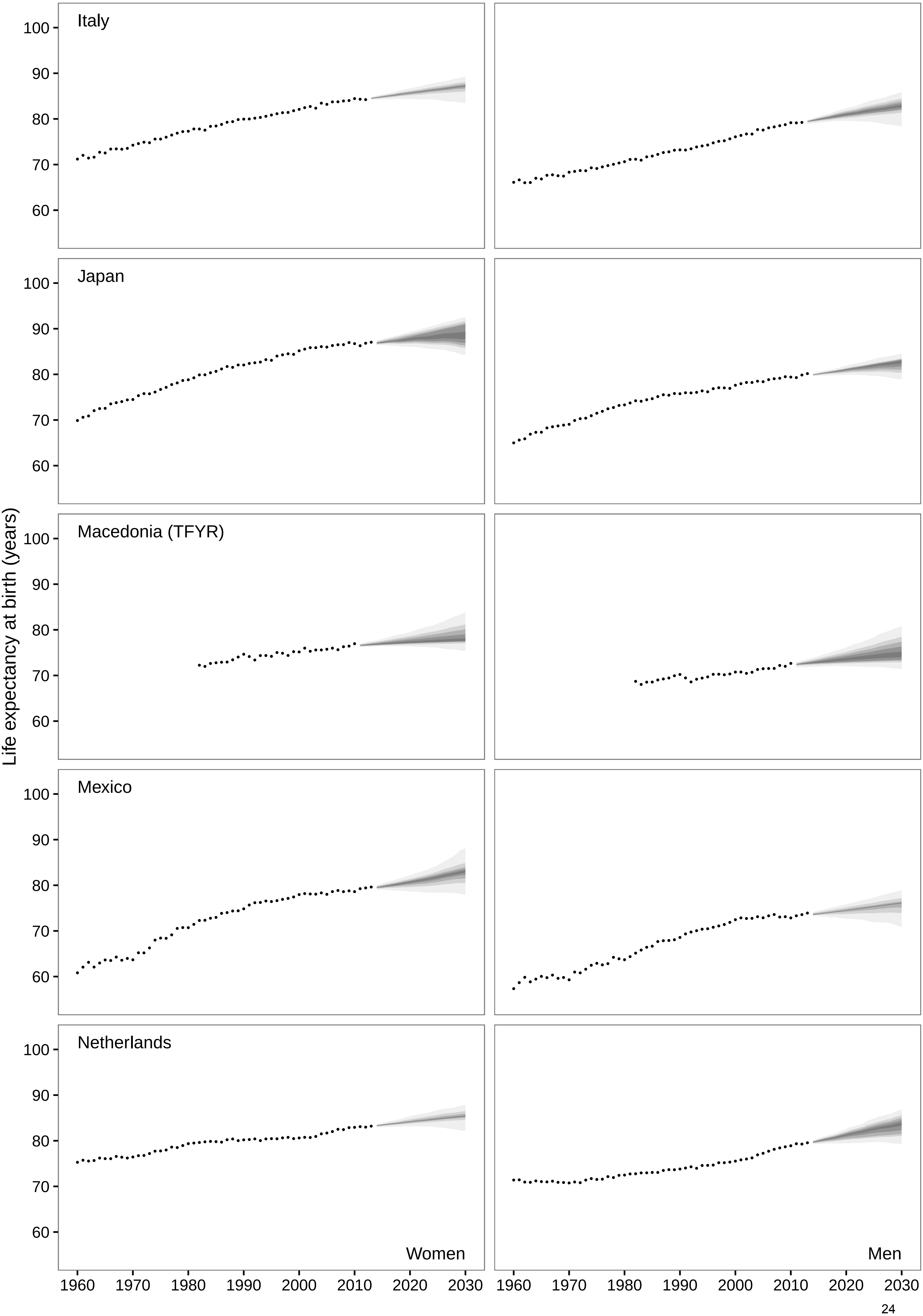
Italy
Japan
Macedonia (TFYR)
Mexico
Netherlands
Life expectancy at birth (years)
Women
Men
100
90
80
70
60
1960
1970
1980
1990
2000
2010
2020
2030

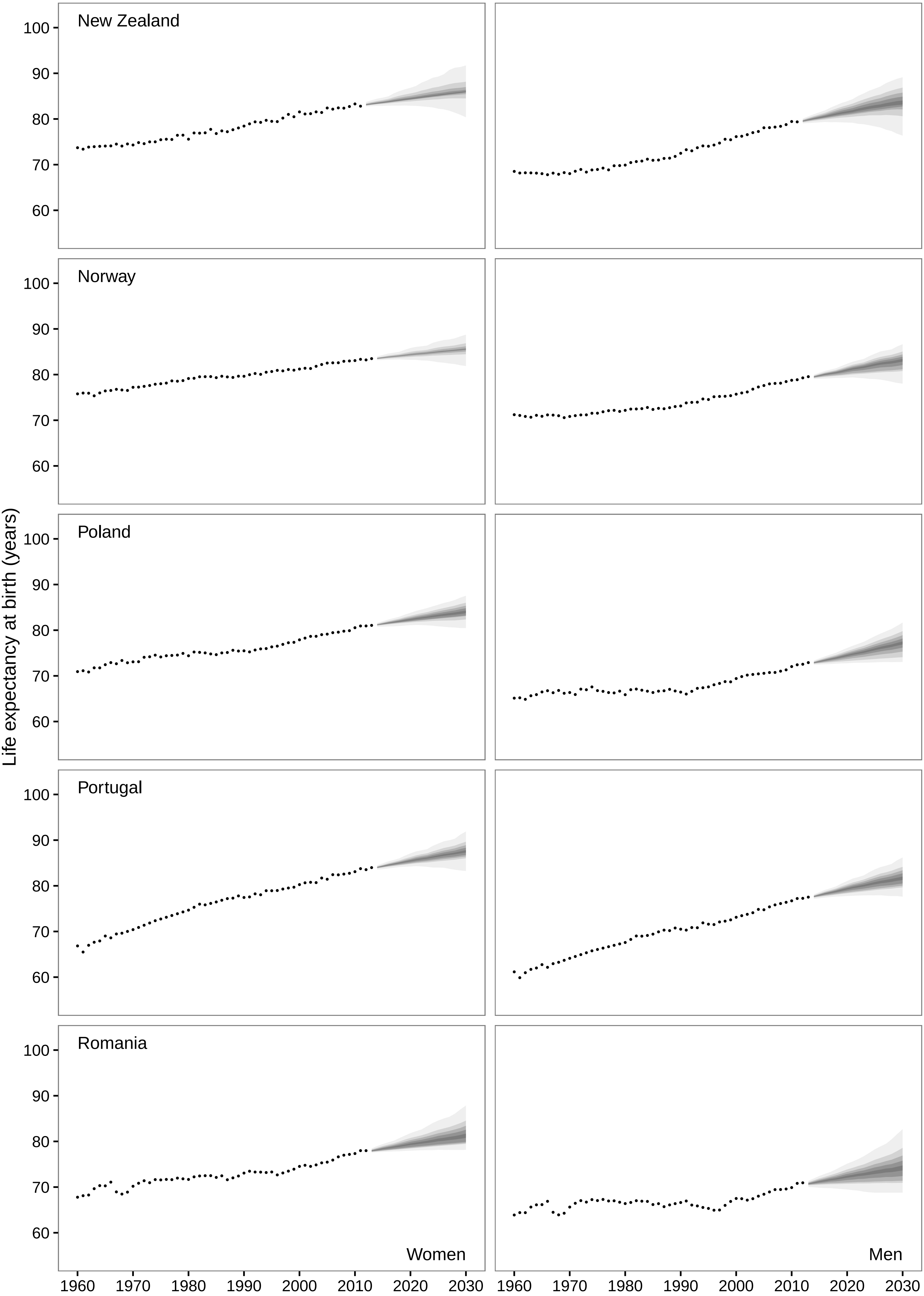
New Zealand
Norway
Poland
Portugal
Romania
Women
Men
Life expectancy at birth (years)
100
90
80
70
60
1960
1970
1980
1990
2000
2010
2020
2030

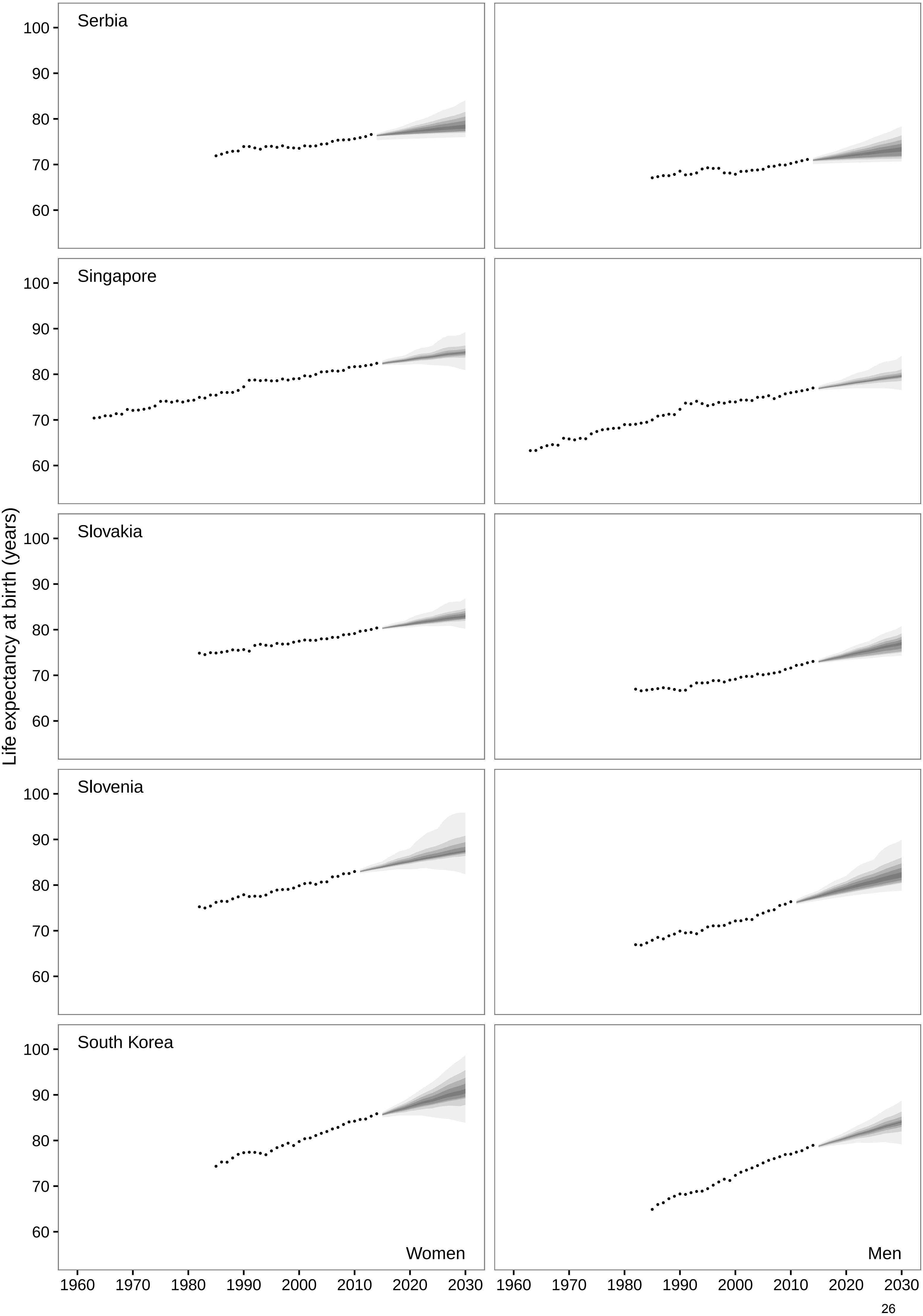

Serbia
Singapore
Slovakia
Slovenia
South Korea
Life expectancy at birth (years)
100
90
80
70
60
Women
Men
1960
1970
1980
1990
2000
2010
2020
2030

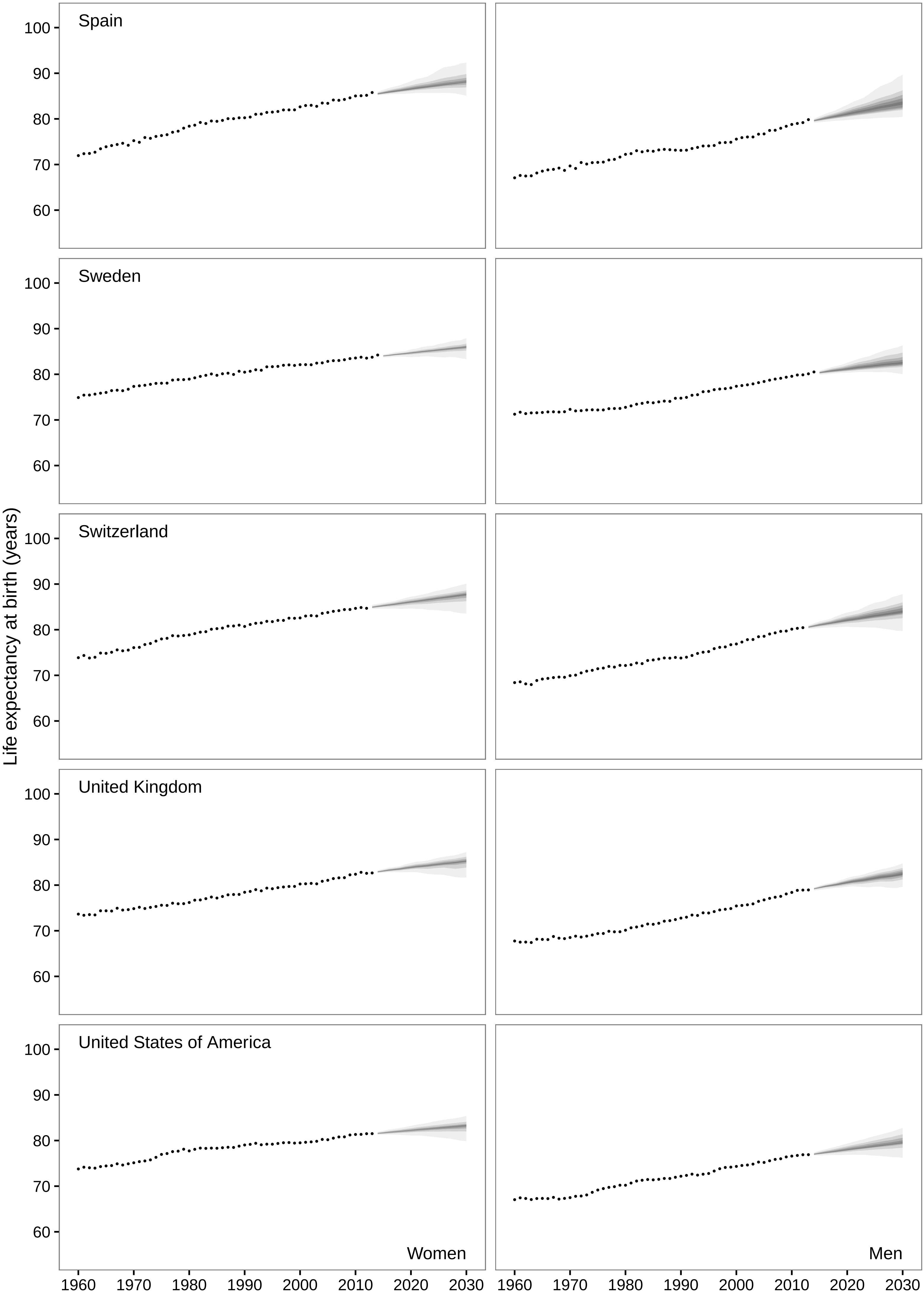
Spain
Sweden
Switzerland
United Kingdom
United States of America
Women
Men
Life expectancy at birth (years)
100
90
80
70
60
1960
1970
1980
1990
2000
2010
2020
2030

Appendix Figure 5: Women's (A) and men's (B) probability of dying before 70 years of age in 2010 and 2030. The left panel shows the posterior distribution of the probability of dying before 70 years of age and its median value for each country. The right panel shows the probability distribution for each country's ranking. See Appendix Table 4 for numerical values.

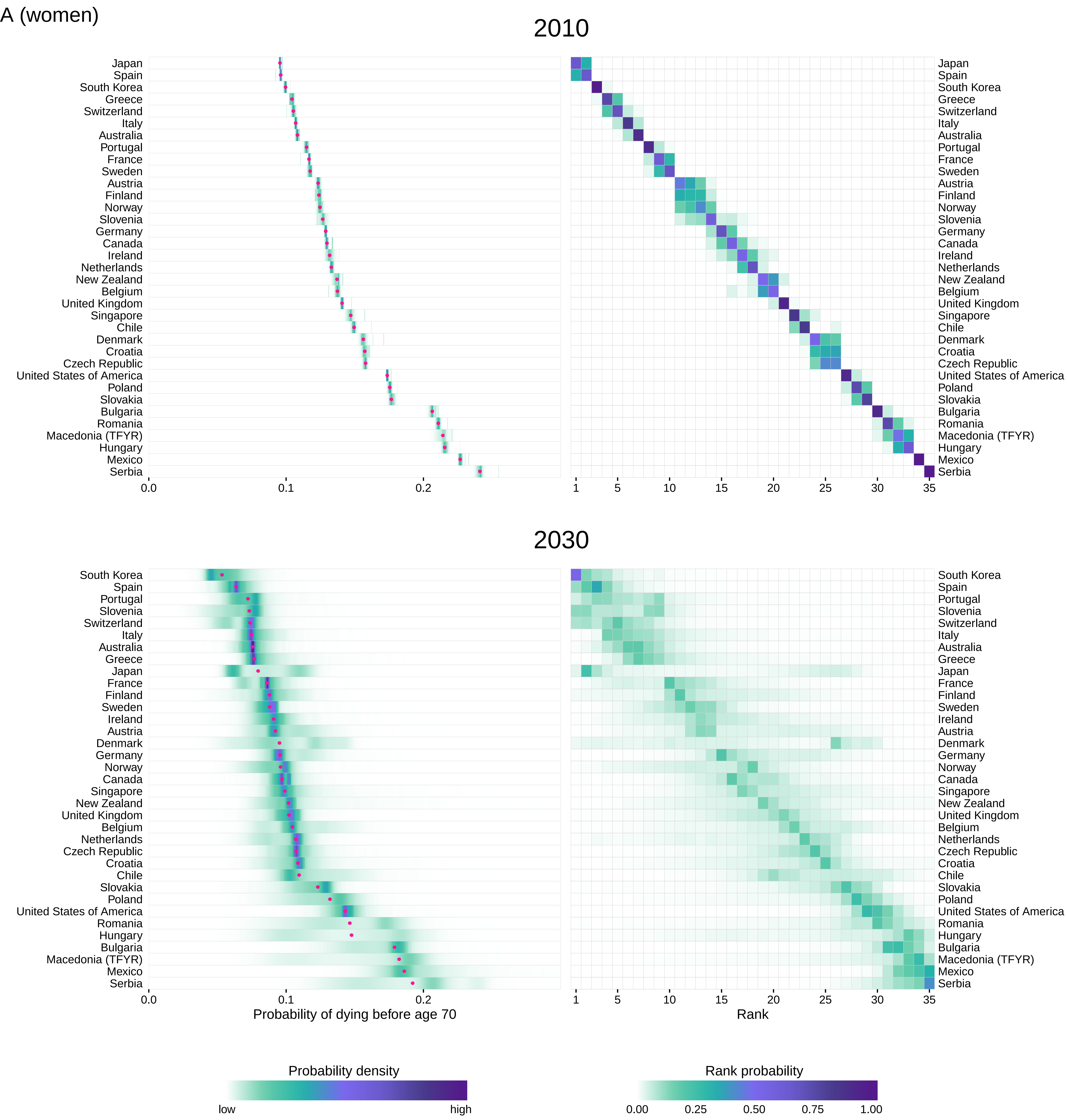
A (women)
2010
Japan
Spain
South Korea
Greece
Switzerland
Italy
Australia
Portugal
France
Sweden
Austria
Finland
Norway
Slovenia
Germany
Canada
Ireland
Netherlands
New Zealand
Belgium
United Kingdom
Singapore
Chile
Denmark
Croatia
Czech Republic
United States of America
Poland
Slovakia
Bulgaria
Romania
Macedonia (TFYR)
Hungary
Mexico
Serbia
2030
South Korea
Spain
Portugal
Slovenia
Switzerland
Italy
Australia
Greece
Japan
France
Finland
Sweden
Ireland
Austria
Denmark
Germany
Norway
Canada
Singapore
New Zealand
United Kingdom
Belgium
Netherlands
Czech Republic
Croatia
Chile
Slovakia
Poland
United States of America
Romania
Hungary
Bulgaria
Macedonia (TFYR)
Mexico
Serbia
0.0
0.1
0.2
1
5
10
15
20
25
30
35
Probability of dying before age 70
Rank
Probability density
low
high
Rank probability
0.00
0.25
0.50
0.75
1.00

B (men)

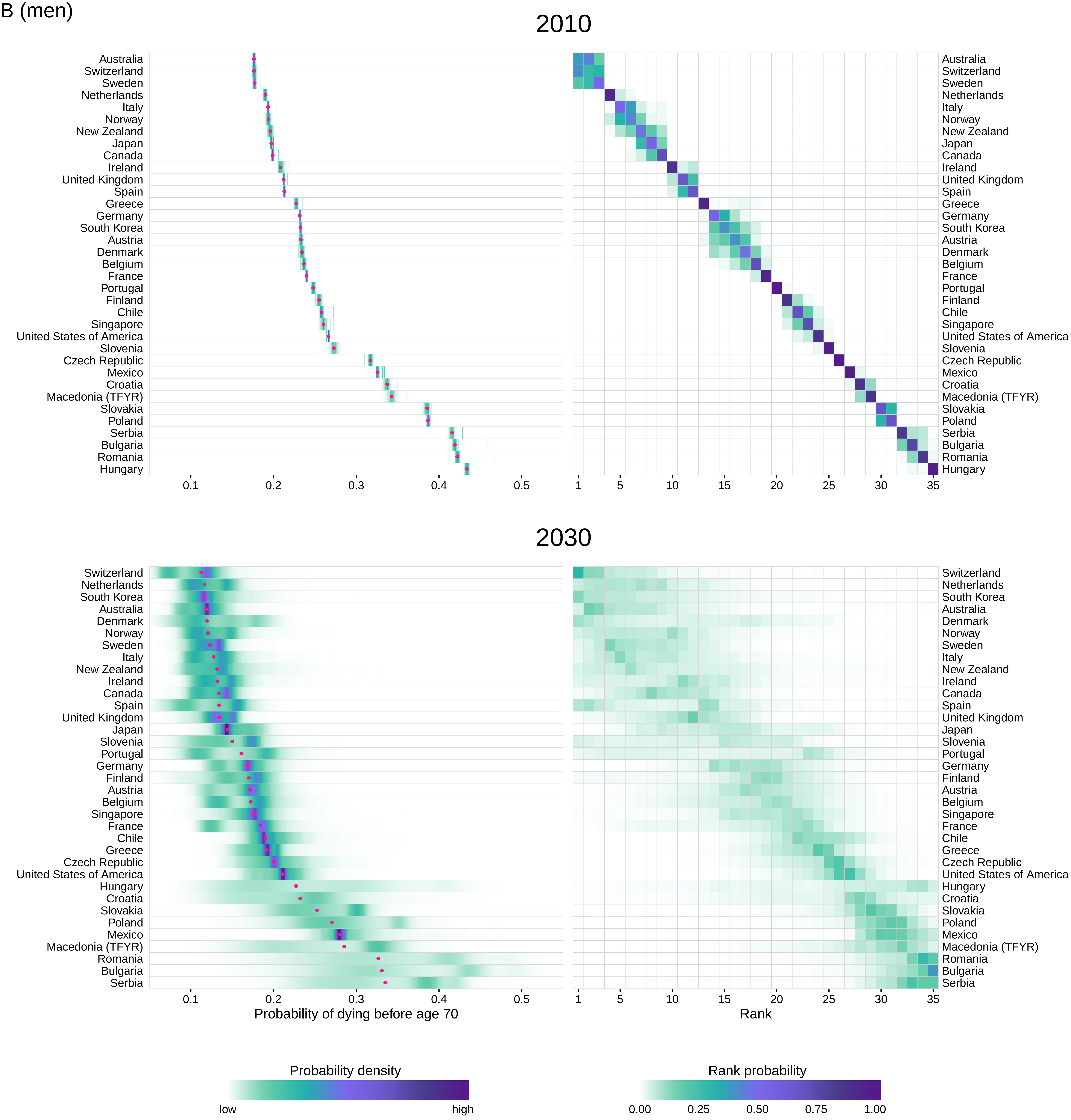
2010
Australia
Switzerland
Sweden
Netherlands
Italy
Norway
New Zealand
Japan
Canada
Ireland
United Kingdom
Spain
Greece
Germany
South Korea
Austria
Denmark
Belgium
France
Portugal
Finland
Chile
Singapore
United States of America
Slovenia
Czech Republic
Mexico
Croatia
Macedonia (TFYR)
Slovakia
Poland
Serbia
Bulgaria
Romania
Hungary
0.1 0.2 0.3 0.4 0.5
1 5 10 15 20 25 30 35
2030
Switzerland
Netherlands
South Korea
Australia
Denmark
Norway
Sweden
Italy
New Zealand
Ireland
Canada
Spain
United Kingdom
Japan
Slovenia
Portugal
Germany
Finland
Austria
Belgium
Singapore
France
Chile
Greece
Czech Republic
United States of America
Hungary
Croatia
Slovakia
Poland
Mexico
Macedonia (TFYR)
Romania
Bulgaria
Serbia
0.1 0.2 0.3 0.4 0.5
Probability of dying before age 70
1 5 10 15 20 25 30 35
Rank
Probability density
low
high
Rank probability
0.00 0.25 0.50 0.75 1.00

Appendix Figure 6: Comparison of projection bias in life expectancy at birth vs projection bias in log-transformed death rates. Each point corresponds to one country and one model.

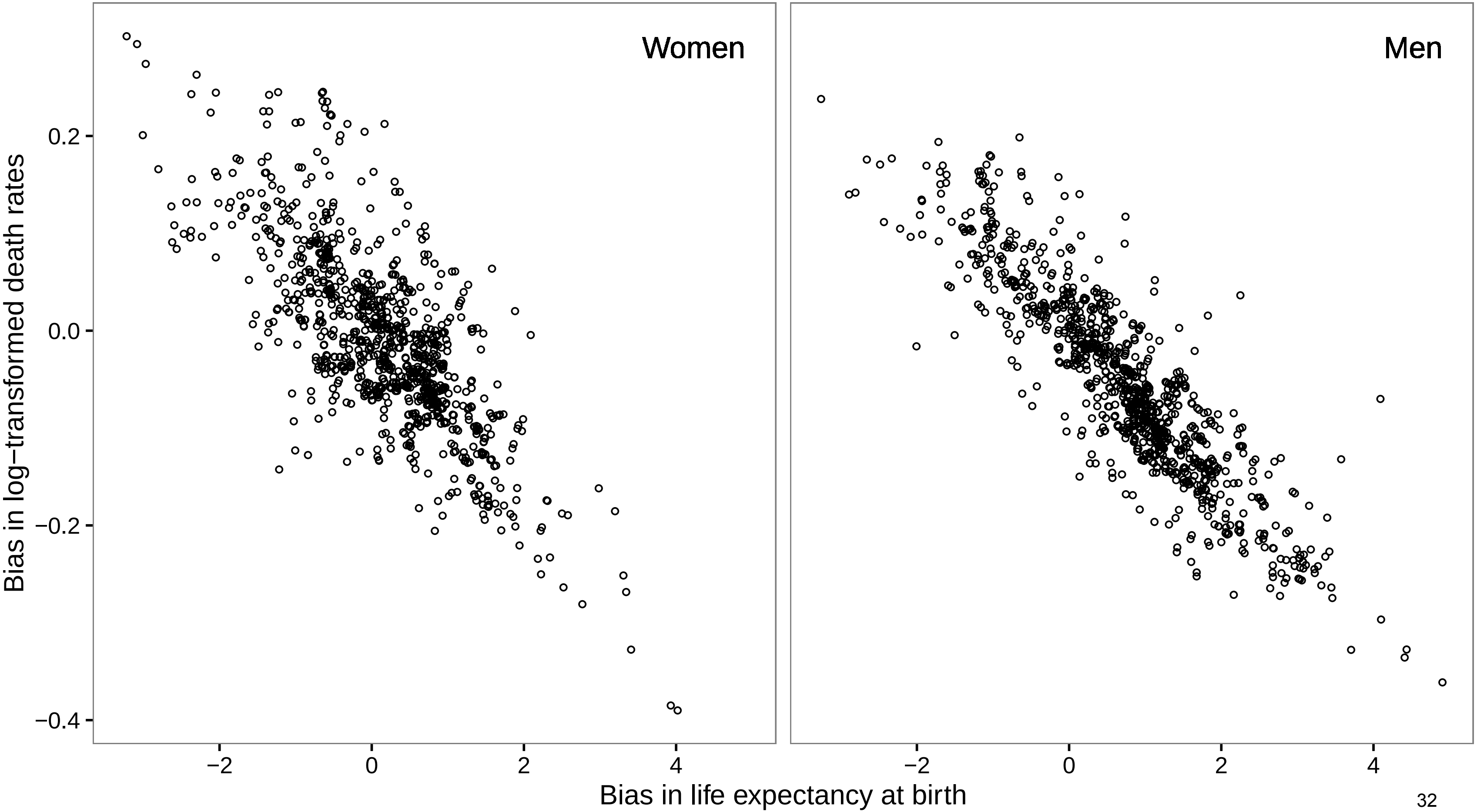
Women
Men
Bias in log−transformed death rates
Bias in life expectancy at birth
0.2
0.0
−0.2
−0.4
−2
0
2
4

Appendix Figure 7: Comparison of projection deviation vs projection bias for life expectancy. Each point corresponds to one country and one model.

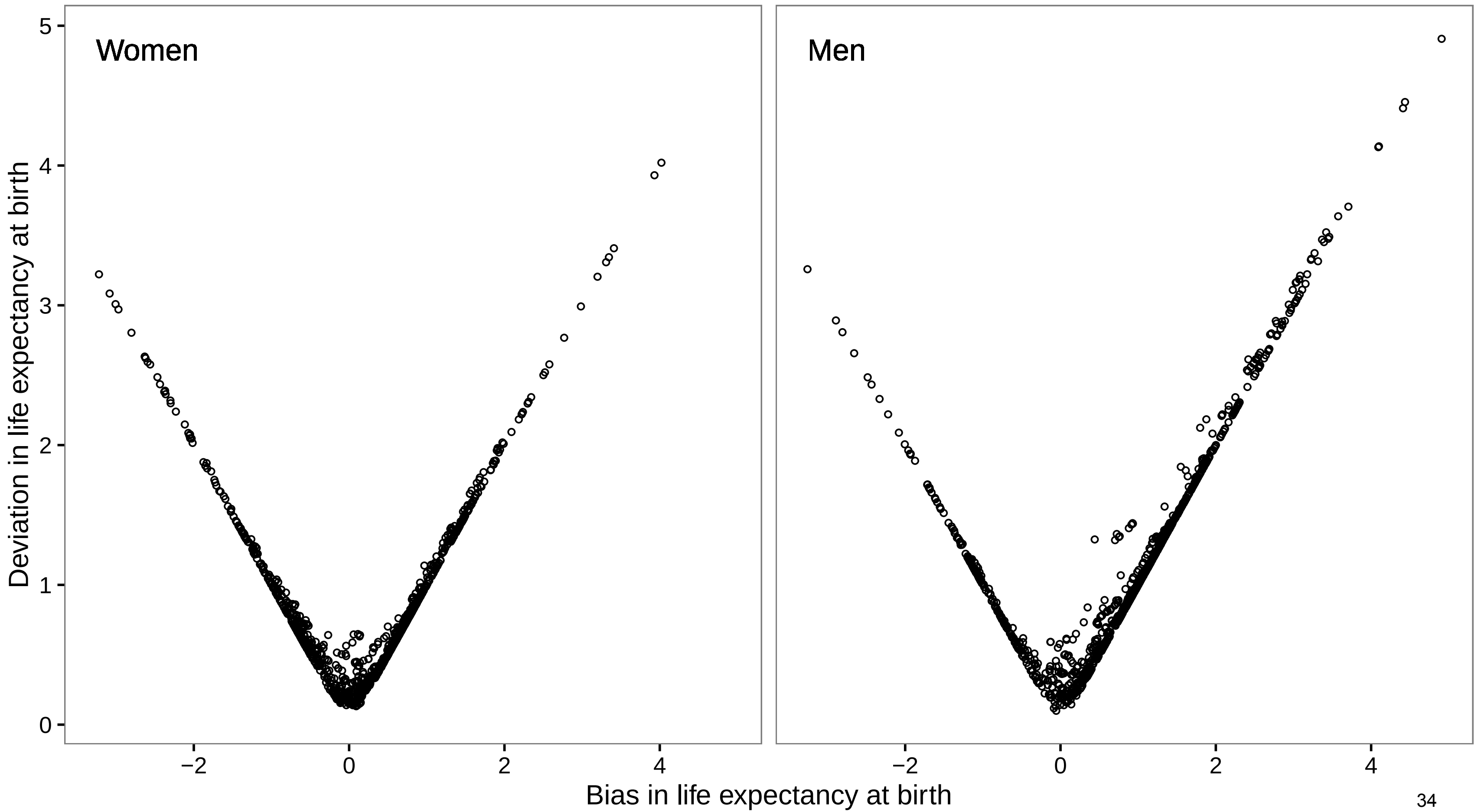
Women
Men
Deviation in life expectancy at birth
Bias in life expectancy at birth
0
1
2
3
4
5
−2
0
2
4

Appendix Table 1: Ninety percent coverage of life expectancy projections in validation analyses. The percentage measures the proportion of observed (but withheld) data points which lie within the 90% credible interval of the BMA projections of life expectancy at birth (over the 1989-2010 validation period, as described in Appendix Text 4).

**Country**	**Men**	**Women**
Australia	95.5%	95.5%
Austria	100%	100%
Belgium	100%	100%
Canada	100%	100%
Chile	90.9%	100%
Denmark	45.5%	100%
Finland	100%	100%
France	100%	72.7%
Hungary	59.1%	59.1%
Ireland	100%	100%
Italy	100%	100%
Japan	54.5%	86.4%
Mexico	100%	100%
Netherlands	100%	100%
New Zealand	86.4%	81.8%
Norway	9.1%	100%
Poland	27.3%	36.4%
Portugal	95.5%	100%
Romania	54.5%	36.4%
Spain	100%	100%
Sweden	100%	100%
Switzerland	90.9%	95.5%
United Kingdom	100%	100%
United States of America	100%	100%
**All countries**	**83.9%**	**90.2%**

Appendix Table 2: Life expectancy at birth in 2010 and 2030.

Country	Men		Women	
	2010	2030	2010	2030
Australia	80.10 (79.89-80.19)	84.00 (80.27-87.95)	84.53 (84.46-84.64)	87.57 (84.86-90.43)
Austria	77.73 (77.50-77.87)	81.40 (78.56-85.40)	83.21 (82.90-83.33)	86.22 (81.65-88.32)
Belgium	77.35 (77.07-77.46)	80.88 (76.38-83.80)	82.76 (82.57-82.87)	85.64 (81.10-87.98)
Bulgaria	70.21 (68.56-70.34)	74.07 (67.88-80.80)	77.33 (76.93-77.45)	78.87 (77.51-84.06)
Canada	79.41 (78.70-79.50)	83.89 (80.01-87.87)	83.94 (83.40-84.04)	87.09 (84.11-90.02)
Chile	76.74 (76.09-76.86)	80.74 (76.23-84.05)	82.95 (81.73-83.08)	86.89 (80.71-90.20)
Croatia	73.10 (72.72-73.27)	77.80 (72.78-85.25)	79.80 (79.50-79.92)	83.19 (80.06-88.92)
Czech Republic	74.36 (74.24-74.51)	79.51 (75.90-81.98)	80.63 (80.46-80.74)	84.42 (80.98-86.93)
Denmark	77.22 (77.06-77.34)	82.53 (78.46-86.36)	81.33 (80.86-81.48)	84.95 (81.10-91.50)
Finland	76.72 (76.47-76.87)	81.05 (78.76-84.56)	83.30 (83.12-83.45)	86.43 (81.27-88.35)
France	77.93 (77.78-78.09)	81.74 (76.89-85.83)	84.86 (84.61-85.15)	88.55 (84.64-91.28)
Germany	77.91 (77.74-77.97)	81.96 (78.54-85.58)	82.81 (82.73-82.86)	85.86 (81.46-87.13)
Greece	77.89 (77.25-78.01)	80.59 (76.90-84.28)	82.63 (82.24-82.75)	84.82 (80.31-88.70)
Hungary	70.65 (70.53-70.75)	78.18 (72.04-85.12)	78.42 (78.31-78.53)	81.74 (78.33-86.91)
Ireland	77.96 (77.70-78.14)	83.22 (78.69-87.74)	82.59 (82.34-82.77)	86.15 (81.70-91.02)
Italy	78.94 (78.50-78.99)	82.82 (78.36-85.92)	84.18 (83.90-84.23)	87.28 (83.45-89.29)
Japan	79.44 (79.24-79.48)	82.75 (78.91-84.61)	86.66 (86.04-86.96)	88.41 (84.22-92.60)
Macedonia (TFYR)	72.29 (71.69-72.54)	74.65 (71.39-80.85)	76.47 (76.23-76.73)	77.83 (75.35-83.88)
Mexico	73.15 (73.00-73.23)	76.15 (70.82-78.95)	78.91 (78.57-78.98)	82.96 (77.89-88.09)
Netherlands	78.91 (78.77-79.01)	83.69 (79.29-86.91)	82.85 (82.74-83.00)	85.39 (82.17-87.89)
New Zealand	79.01 (78.82-79.20)	83.59 (76.35-89.14)	82.80 (82.66-83.00)	85.96 (80.36-91.73)
Norway	78.69 (78.52-78.85)	83.16 (78.01-86.65)	83.08 (82.94-83.23)	85.55 (81.87-88.75)
Poland	71.86 (71.79-71.93)	77.21 (73.04-81.68)	80.40 (80.32-80.47)	84.01 (80.47-87.55)
Portugal	76.71 (76.53-76.81)	81.68 (77.62-86.21)	83.14 (82.98-83.23)	87.52 (83.24-91.90)
Romania	70.12 (69.98-70.21)	74.21 (68.78-82.68)	77.46 (77.18-77.55)	81.24 (78.15-87.85)
Serbia	70.25 (69.91-70.39)	73.37 (70.67-78.37)	75.71 (75.22-75.83)	78.27 (75.99-84.07)
Singapore	75.87 (75.67-76.05)	79.57 (76.56-84.04)	81.54 (81.12-81.71)	84.81 (80.92-89.27)
Slovakia	71.62 (71.49-71.78)	76.98 (74.31-80.85)	79.25 (79.14-79.38)	82.92 (80.21-86.87)
Slovenia	75.88 (75.62-76.11)	82.26 (78.76-89.88)	82.68 (82.48-82.90)	87.42 (82.38-95.86)
South Korea	77.11 (76.86-77.19)	84.07 (79.17-88.74)	84.23 (84.13-84.30)	90.82 (83.91-98.70)
Spain	78.66 (78.59-78.72)	83.47 (80.45-89.72)	84.83 (84.60-84.89)	88.07 (85.08-92.33)
Sweden	79.55 (79.39-79.66)	82.52 (80.03-86.35)	83.49 (83.39-83.58)	85.98 (83.34-87.94)
Switzerland	80.01 (79.87-80.14)	83.95 (79.71-87.79)	84.59 (84.47-85.08)	87.70 (83.53-90.09)
United Kingdom	78.34 (78.25-78.39)	82.47 (79.65-84.79)	82.32 (81.96-82.37)	85.25 (81.66-87.23)
United States of America	76.52 (76.45-76.55)	79.51 (76.22-82.78)	81.24 (81.01-81.27)	83.32 (79.83-85.39)

Appendix Table 3: Life expectancy at age 65 years

Country	Men		Women	
	2010	2030	2010	2030
Australia	19.36 (19.05-19.44)	22.19 (18.73-26.01)	22.32 (22.25-22.41)	24.60 (22.27-27.56)
Austria	17.67 (17.44-17.78)	20.03 (17.19-23.67)	21.09 (20.75-21.19)	23.43 (19.40-25.49)
Belgium	17.46 (17.07-17.55)	19.83 (15.76-22.23)	21.07 (20.85-21.16)	23.31 (18.74-25.66)
Bulgaria	13.64 (12.88-13.73)	15.87 (12.58-21.99)	16.90 (16.46-16.98)	17.85 (16.83-22.64)
Canada	19.05 (18.19-19.12)	22.57 (18.41-26.47)	22.18 (21.62-22.26)	24.62 (22.16-27.93)
Chile	17.90 (17.19-18.02)	20.14 (15.97-23.84)	21.79 (20.58-21.91)	24.50 (19.67-28.26)
Croatia	14.47 (14.03-14.60)	17.19 (12.83-25.18)	18.04 (17.70-18.14)	20.56 (17.82-26.92)
Czech Republic	15.32 (15.23-15.49)	18.71 (15.59-20.80)	18.82 (18.66-18.92)	21.79 (18.58-24.13)
Denmark	16.95 (16.83-17.06)	20.42 (16.64-24.15)	19.64 (19.15-19.76)	22.22 (18.33-28.88)
Finland	17.36 (16.99-17.48)	19.98 (17.30-23.05)	21.30 (21.03-21.42)	23.81 (18.54-25.66)
France	18.62 (18.42-18.78)	21.47 (17.83-24.69)	23.02 (22.73-23.18)	26.05 (22.43-29.01)
Germany	17.73 (17.57-17.78)	20.66 (17.79-23.94)	20.78 (20.69-20.82)	23.14 (19.49-24.44)
Greece	18.06 (17.41-18.15)	20.11 (15.86-24.05)	20.15 (19.63-20.26)	21.76 (17.84-26.35)
Hungary	14.06 (13.97-14.14)	17.53 (13.88-24.68)	18.07 (17.96-18.15)	20.03 (16.60-24.63)
Ireland	17.52 (17.31-17.68)	21.69 (17.18-26.12)	20.63 (20.40-20.78)	23.41 (18.70-28.47)
Italy	18.04 (17.65-18.08)	20.63 (16.38-23.51)	21.75 (21.42-21.79)	24.12 (20.93-26.27)
Japan	18.79 (18.57-18.87)	21.07 (17.70-22.98)	24.34 (23.64-24.69)	25.92 (21.93-29.56)
Macedonia (TFYR)	13.61 (13.26-13.77)	14.53 (12.50-19.90)	15.51 (15.22-15.70)	16.00 (14.41-22.42)
Mexico	16.94 (16.84-17.01)	18.33 (15.56-21.99)	19.58 (19.34-19.64)	22.75 (18.63-28.90)
Netherlands	17.72 (17.53-17.80)	21.47 (17.78-24.46)	20.94 (20.83-21.10)	22.99 (19.66-25.00)
New Zealand	18.80 (18.60-18.96)	22.52 (15.88-27.68)	21.24 (21.11-21.42)	23.68 (19.27-30.19)
Norway	17.80 (17.65-17.94)	21.04 (16.77-24.33)	20.92 (20.80-21.04)	22.86 (18.94-25.73)
Poland	14.92 (14.81-14.97)	18.08 (14.85-22.36)	19.33 (19.20-19.38)	22.02 (17.95-25.34)
Portugal	17.27 (17.15-17.35)	20.21 (16.53-24.26)	20.97 (20.84-21.06)	24.39 (20.13-28.61)
Romania	13.95 (13.87-14.02)	15.83 (11.78-23.62)	17.13 (16.94-17.19)	19.39 (16.82-25.63)
Serbia	12.94 (12.72-13.03)	14.37 (12.57-18.37)	15.22 (14.88-15.30)	17.09 (15.04-23.07)
Singapore	15.60 (15.44-15.74)	17.92 (15.42-22.69)	19.55 (19.22-19.71)	21.96 (18.63-27.20)
Slovakia	14.09 (13.96-14.19)	17.15 (15.10-20.63)	18.01 (17.91-18.11)	20.79 (18.39-24.87)
Slovenia	16.47 (16.26-16.68)	20.42 (17.49-28.06)	20.60 (20.42-20.77)	24.29 (19.79-33.09)
South Korea	17.03 (16.83-17.11)	21.96 (17.61-26.74)	21.82 (21.71-21.90)	27.50 (20.31-35.72)
Spain	18.24 (18.17-18.37)	21.19 (18.13-26.93)	22.33 (22.04-22.39)	24.77 (21.66-29.13)
Sweden	18.26 (18.09-18.34)	20.42 (18.42-24.19)	21.08 (21.00-21.22)	23.02 (20.76-25.10)
Switzerland	18.91 (18.80-19.01)	21.52 (17.33-25.04)	22.21 (22.11-22.67)	24.62 (20.39-26.90)
United Kingdom	18.07 (18.00-18.11)	20.86 (17.22-23.02)	20.62 (20.23-20.66)	22.73 (18.75-24.70)
United States of America	18.14 (17.90-18.16)	20.12 (16.62-22.87)	20.62 (20.32-20.64)	22.00 (18.62-24.12)

Appendix Table 4: Probability of dying before 70 years of age

Country	Men		Women	
	2010	2030	2010	2030
Australia	0.18 (0.17-0.18)	0.12 (0.08-0.16)	0.11 (0.11-0.11)	0.08 (0.06-0.10)
Austria	0.23 (0.23-0.24)	0.17 (0.11-0.22)	0.12 (0.12-0.13)	0.09 (0.07-0.14)
Belgium	0.24 (0.23-0.24)	0.17 (0.11-0.23)	0.14 (0.13-0.14)	0.10 (0.07-0.15)
Bulgaria	0.42 (0.42-0.46)	0.33 (0.20-0.49)	0.21 (0.20-0.21)	0.18 (0.12-0.21)
Canada	0.20 (0.20-0.21)	0.13 (0.09-0.17)	0.13 (0.13-0.13)	0.10 (0.08-0.12)
Chile	0.26 (0.26-0.27)	0.19 (0.16-0.27)	0.15 (0.15-0.16)	0.11 (0.09-0.17)
Croatia	0.34 (0.33-0.35)	0.23 (0.12-0.38)	0.16 (0.15-0.16)	0.11 (0.07-0.18)
Czech Republic	0.32 (0.31-0.32)	0.20 (0.15-0.28)	0.16 (0.16-0.16)	0.11 (0.08-0.14)
Denmark	0.23 (0.23-0.24)	0.12 (0.07-0.20)	0.16 (0.15-0.17)	0.10 (0.05-0.14)
Finland	0.25 (0.25-0.26)	0.17 (0.09-0.21)	0.12 (0.12-0.13)	0.09 (0.06-0.12)
France	0.24 (0.24-0.24)	0.18 (0.11-0.24)	0.12 (0.11-0.12)	0.09 (0.06-0.11)
Germany	0.23 (0.23-0.23)	0.17 (0.12-0.22)	0.13 (0.13-0.13)	0.10 (0.08-0.14)
Greece	0.23 (0.22-0.24)	0.19 (0.15-0.26)	0.10 (0.10-0.11)	0.08 (0.07-0.13)
Hungary	0.43 (0.43-0.44)	0.23 (0.11-0.42)	0.22 (0.21-0.22)	0.15 (0.08-0.21)
Ireland	0.21 (0.20-0.22)	0.13 (0.10-0.21)	0.13 (0.13-0.14)	0.09 (0.07-0.13)
Italy	0.19 (0.19-0.20)	0.13 (0.09-0.19)	0.11 (0.11-0.11)	0.07 (0.06-0.11)
Japan	0.20 (0.20-0.20)	0.14 (0.12-0.20)	0.10 (0.09-0.10)	0.08 (0.06-0.12)
Macedonia (TFYR)	0.34 (0.34-0.36)	0.28 (0.15-0.38)	0.21 (0.21-0.22)	0.18 (0.09-0.24)
Mexico	0.33 (0.32-0.33)	0.28 (0.24-0.38)	0.23 (0.23-0.23)	0.19 (0.15-0.27)
Netherlands	0.19 (0.19-0.19)	0.12 (0.09-0.17)	0.13 (0.13-0.13)	0.11 (0.07-0.13)
New Zealand	0.20 (0.19-0.20)	0.13 (0.09-0.22)	0.14 (0.13-0.14)	0.10 (0.08-0.19)
Norway	0.19 (0.19-0.20)	0.12 (0.09-0.20)	0.12 (0.12-0.13)	0.10 (0.06-0.12)
Poland	0.39 (0.38-0.39)	0.27 (0.18-0.37)	0.18 (0.17-0.18)	0.13 (0.08-0.16)
Portugal	0.25 (0.25-0.25)	0.16 (0.09-0.23)	0.11 (0.11-0.12)	0.07 (0.05-0.11)
Romania	0.42 (0.42-0.43)	0.33 (0.17-0.49)	0.21 (0.21-0.22)	0.15 (0.08-0.20)
Serbia	0.42 (0.41-0.43)	0.33 (0.21-0.42)	0.24 (0.24-0.25)	0.19 (0.12-0.24)
Singapore	0.26 (0.26-0.27)	0.18 (0.12-0.23)	0.15 (0.14-0.16)	0.10 (0.08-0.17)
Slovakia	0.39 (0.38-0.39)	0.25 (0.17-0.32)	0.18 (0.17-0.18)	0.12 (0.08-0.15)
Slovenia	0.27 (0.27-0.28)	0.15 (0.08-0.20)	0.13 (0.12-0.13)	0.07 (0.04-0.11)
South Korea	0.23 (0.23-0.24)	0.12 (0.09-0.19)	0.10 (0.10-0.10)	0.05 (0.04-0.08)
Spain	0.21 (0.21-0.21)	0.13 (0.07-0.18)	0.10 (0.09-0.10)	0.06 (0.05-0.08)
Sweden	0.18 (0.17-0.18)	0.12 (0.09-0.15)	0.12 (0.12-0.12)	0.09 (0.07-0.11)
Switzerland	0.18 (0.17-0.18)	0.11 (0.06-0.16)	0.11 (0.10-0.11)	0.07 (0.04-0.10)
United Kingdom	0.21 (0.21-0.21)	0.13 (0.09-0.16)	0.14 (0.14-0.15)	0.10 (0.07-0.12)
United States of America	0.27 (0.26-0.27)	0.21 (0.16-0.26)	0.17 (0.17-0.18)	0.14 (0.12-0.17)

Appendix Table 5: Numbered list of ensemble models. See Appendix Text 2 for detailed descriptions of each model. Model numbers in Appendix Figures 1 and 2 refer to the numbers in the first column of this table.

**Model number**	**Model category**		**Version**	
			**Age terms**	**Cohort terms**
1	Age-time		IID[*]	
2			RW1[**]	
3	Age-time-cohort		IID	RW1
4			RW1	RW1
5	Piecewise linear	Knot at -6 years	IID	
6			RW1	
7			IID	RW1
8			RW1	RW1
9		Knot at -10 years	IID	
10			RW1	
11			IID	RW1
12			RW1	RW1
13	Weighted likelihood		IID	
14			RW1	
15			IID	RW1
16			RW1	RW1
17	Lee-Carter	1 PC[†]		
18		2 PC		
19		3 PC		
20		4 PC		
21		5 PC		

[*] IID: independent identically distributed
[**] RW1: random walk order 1
[†] PC: principal component

Appendix Table 6: Total variance of the projected life expectancy in 2030 and the proportion of total variance that is due to between-model variance, by country and sex. The total variance of life expectancy under the model average is the sum of the weighted average of the life expectancy variance under each model ("within-model") and the weighted variance of the life expectancy mean under each model ("between-model").[21, 22] The larger the proportion, the higher the share of projection uncertainty that is due to uncertainty in model choice.

Country	Men		Women	
	**Total variance**	**Between model as a proportion of total variance**	**Total variance**	**Between model as a proportion of total variance**
Australia	3.82	18%	2.45	8%
Austria	2.98	46%	2.82	59%
Belgium	3.69	58%	3.22	49%
Bulgaria	10.48	50%	3.08	41%
Canada	4.69	42%	2.66	18%
Chile	4.12	22%	6.07	40%
Croatia	10.47	14%	5.12	12%
Czech Republic	2.39	21%	2.02	42%
Denmark	5.51	51%	7.67	37%
Finland	2.42	17%	4.13	57%
France	4.93	66%	2.53	41%
Germany	3.07	53%	1.96	61%
Greece	3.54	50%	4.13	68%
Hungary	12.27	54%	5.38	43%
Ireland	6.70	42%	5.81	30%
Italy	3.58	38%	2.47	30%
Japan	2.78	52%	7.53	80%
Macedonia (TFYR)	7.95	43%	6.10	29%
Mexico	3.95	47%	6.93	16%
Netherlands	4.72	55%	1.78	32%
New Zealand	12.04	42%	8.21	25%
Norway	4.99	53%	2.85	26%
Poland	5.56	49%	3.67	37%
Portugal	5.50	38%	4.72	40%
Romania	13.36	27%	6.59	32%
Serbia	5.03	58%	5.07	55%
Singapore	3.15	7%	4.35	18%
Slovakia	3.47	52%	2.72	29%
Slovenia	9.34	38%	12.37	19%
South Korea	5.20	41%	13.93	62%
Spain	6.00	23%	3.60	15%
Sweden	2.71	43%	1.34	24%
Switzerland	4.36	31%	3.24	26%
United Kingdom	1.77	41%	2.03	45%
United States of America	2.54	42%	1.86	28%
